# Gastroenterological disorders and hepatic disease in adults with cerebral palsy: A systematic review

**DOI:** 10.1111/dmcn.70034

**Published:** 2025-10-30

**Authors:** Christina M. Marciniak, Jennifer M. Ryan, Alejandra Camacho‐Soto, Emily Capellari, Jessica Burke, Maram Sofiany, Zoë Post, Eric C. Sung, Michael D. Brown, the Adult Cerebral Palsy Clinical Practice Guidelines Working Group, Jennifer M Ryan, Rachel Byrne, Emily Capellari, Donna Riccio Omichinski, Mark Peterson, Jan Willem Gorter, Ashley Harris Whaley, Christine Imms, Christina M. Marciniak

**Affiliations:** ^1^ Department of Physical Medicine Northwestern University, Feinberg Medical School Chicago IL USA; ^2^ Department of Neurology Northwestern University, Feinberg Medical School Chicago IL USA; ^3^ CP‐Life Research Centre, School of Physiotherapy Royal College of Surgeons in Ireland Dublin Ireland; ^4^ Department of Physical Medicine and Rehabilitation The University of Kansas Medical Center Kansas City KS USA; ^5^ Taubman Health Sciences Library University of Michigan Ann Arbor MI USA; ^6^ Section of Digestive Diseases and Nutrition Rush University Chicago IL USA; ^7^ Division of Regenerative and Reconstructive Sciences University of California Los Angeles Los Angeles CA USA

## Abstract

**Aim:**

To describe the incidence, prevalence, and prognostic factors for gastroenterological disorders and hepatic disease in adults with cerebral palsy (CP), and to examine the effectiveness of any screening or interventions.

**Method:**

Six databases were searched for articles published in any language since 1990 meeting eligibility criteria, defined for each of five objectives. Two independent reviewers screened study titles, abstracts, and full texts for inclusion.

**Results:**

Thirty‐two reports of 30 unique samples, including 10 to 16 818 adults, were identified. Twenty‐five reported prevalence of at least one of the following: gastroesophageal reflux disease (GERD) (prevalence 3%–42%; seven studies), constipation (4%–67%; seven studies), dysphagia (6%–77%; 12 studies), fecal incontinence (6%–29%; three studies), dental/oral cavity disorders (25%–53%; five studies), and aggregated hepatic diseases (1%–6%; seven studies). The prevalences of GERD, dysphagia, and hepatic disease were higher in adults with CP than in those without. The prevalence of fecal incontinence was greater in people classified as having CP in higher Gross Motor Function Classification System levels. No incidence studies were identified. Four intervention studies addressing oral/dental health or dysphagia were found, but certainty of evidence was low to very low.

**Interpretation:**

The prevalence of specific gastroenterological disorders and hepatic disease varies across studies in adults with CP. Evidence for intervention efficacy in their management is of very low quality to absent.

## Graphical abstract

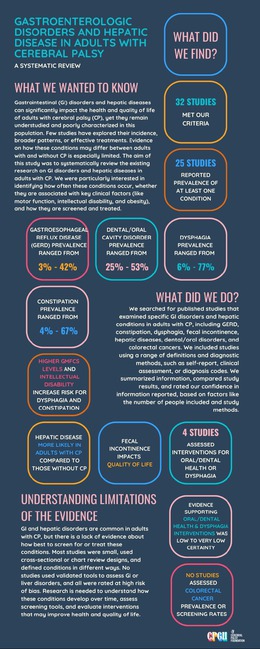

**Abbreviations**
GERD: gastroesophageal reflux disease
RCT: randomized controlled trial

**What this paper adds**

- Prevalence of gastroesophageal reflux disease (GERD), dysphagia, and hepatic disease is higher in adults with cerebral palsy.
- Prevalence of GERD and fecal incontinence is higher in non‐ambulatory adults.
- Risk factors for dysphagia are intellectual disability and worse gross motor function.
- Prevalence of constipation is higher in those with intellectual disability and ataxic/dyskinetic subtypes.
- Targeted education and demonstration interventions may improve dental and oral health measures.

Although the definition of cerebral palsy (CP) has characteristically emphasized impairments in movement and posture, the attributes ascribed to the phenotype of CP have recently been updated to emphasize that manifestations are heterogenous, and, although present early in life, remain lifelong.^1^, ^2^ Indeed, heterogenous findings have been reported both for the characteristic neurological impairments as well as for other organ system involvement.^1^ These impairments can significantly affect life participation for an individual with CP.^1^ Further, the recently updated definition of the CP condition emphasizes that, while features manifest early in life because of non‐degenerative brain dysfunction, resultant impairments may change with age.^1^ Thus, it is important to characterize primary and secondary issues across the entire lifespan, as there may be differences in the features and their impact on individuals with CP over time.

The gastroenterological system (hereafter referred to as the gastrointestinal tract owing to its more conventional use as terminology in the literature) is frequently implicated in children with CP and the cause of many non‐neurological symptoms. Gastrointestinal tract symptoms were identified in 92% of children followed in one neurology clinic,^3^ while another study of non‐ambulatory children identified symptoms of dysphagia, gastroesophageal reflux disease (GERD), or constipation in 82.5%, 40.0%, and 60.0% of children respectively.^4^

Bidirectional communication between the central nervous system and the enteric system, known as the gut–brain axis, is necessary for optimal gut function and integration of gastrointestinal tract processes.^5^ Disruption can affect motility, intestinal secretions, and sensation in the viscera leading to disorders of brain–gut interaction. Gastrointestinal tract dysmotility from central regulation probably contributes to high rates of chronic constipation and GERD in children with CP.^3^, ^6^, ^7^ Gut dysbiosis and abnormal immune responses have recently emerged as potential contributors to constipation and altered immune function in these children, but there has been little study as to their role in gastrointestinal tract symptoms, either in children or in adults.^8^, ^9^, ^10^ Further, liver homeostasis, repair, and regeneration have been found to be influenced by the central nervous system. It has also been identified that there is directional communication from the liver to the central nervous system through hepatokines and other metabolites.^11^ However, to the best of our knowledge, this finding has not been directly studied in children and adults with CP, but rather in adults with other liver disorders. Nevertheless, this indicates that the central nervous system abnormalities found in those with CP may possibly contribute directly to the chronic gastrointestinal tract and hepatic conditions encountered in both children and adults.

For adults with CP, gastrointestinal tract problems and their treatment have not been fully characterized. In a recent systematic review and meta‐analysis describing the prevalence of 53 chronic conditions in adults with CP,^12^ the prevalence of constipation, GERD, bowel incontinence, and hepatic disease was reported, but among these four, only hepatic disease had a higher prevalence in adults with CP than those without, after adjusted analyses.^12^ In addition, the authors did not find studies reporting incidence for any of these gastrointestinal tract disorders or hepatic disease.^12^ A matched cohort study using practice data from England that aggregated information on four cancer types, one of which was colorectal cancer, concluded overall cancer mortality rate was not increased in adulthood.^13^ This is in contrast to an earlier study that reported digestive disease cancers were twice as likely to contribute to mortality over the lifespan of individuals with CP compared with those without it.^14^ Given that colorectal cancer is the second most common cause of cancer deaths in US adults, and adults worldwide, it is critical to understand whether rates differ in adults with CP.

The consequences of many gastrointestinal tract disorders are significant, as they have been shown to contribute to high respiratory morbidity, and respiratory diseases are a cause of heightened mortality in adults with CP.^13^, ^15^ Such disorders include GERD and its contributions to aspiration and dysphagia. Dental/oral cavity disorders also affect dysphagia, aspiration, and nutrition. The oral cavity is considered the upper part of the gastrointestinal tract, and mouth and dental structures are frequently affected by more distal gastrointestinal tract disorders. A recent mapping of systematic reviews concerning adults with CP concluded that most reviews were focused in the areas of cardiovascular and respiratory health or movement‐related functions.^16^ Study of gastrointestinal tract disorders and hepatic diseases is imperative to address gaps in this area, in order to inform clinical practice, support guideline development, optimize long‐term outcomes, and reduce morbidity and mortality for adults with CP.

Therefore, this study was undertaken to review the evidence related to specific gastrointestinal tract disorders and hepatic diseases in adults with CP. Pancreatic diseases or disorders are not explored in this review as they will be discussed in a separate review of cardiometabolic diseases, because the literature on pancreatic‐related disorders in adults with CP largely focuses on diabetes mellitus.

The aim of this study was to systematically review the evidence related to gastrointestinal tract disorders and hepatic diseases in adults with CP. The five objectives of this review were the following. (1) Describe the incidence and prevalence of specific gastrointestinal tract disorders and hepatic diseases among adults with CP, and compared with the general population. (2) Investigate the association between ambulatory status (as a measure of motor severity), the subtypes of CP, intellectual disability, and obesity with the incidence and prevalence of gastrointestinal tract disorders and hepatic diseases among adults with CP. (3) Identify screening tools that have been used to assess the presence, the type, and/or the severity of gastrointestinal tract disorders and hepatic diseases in adults with CP, and investigate the validity and feasibility of these tools in adults with CP. (4) Examine the effectiveness of interventions used for the treatment of the specific gastrointestinal tract disorders and hepatic diseases in adults with CP. (5) Examine the efficacy of screening for colorectal cancers and hepatic diseases in adults with CP.

## METHOD

This review was part of a suite of systematic reviews conducted to inform development of a clinical guideline for the medical care of adults with CP. The reviews were conducted on different domains of health; one protocol was published on the Open Science Framework (https://doi.org/10.17605/OSF.IO/RMXUF) and adapted for each domain. The methods were guided by the JBI Manual for Evidence Synthesis.^17^

Results are reported using the Preferred Reporting Items for Systematic Reviews and Meta‐analyses (PRISMA) and Meta‐Analyses of Observational Studies in Epidemiology (MOOSE) statements.

### Literature search

An experienced information specialist developed and conducted comprehensive searches using the online databases PubMed, Embase, Cochrane CENTRAL, Cochrane Database of Systematic Reviews, CINAHL, and PsycINFO. A single search was conducted in each database to identify studies for all objectives. Reference lists of relevant systematic reviews and included studies were searched for additional articles. An example search strategy for PubMed is in Appendix S1.

### Eligibility criteria

Research questions were developed by the research team consisting of researchers, clinical experts in the field of CP, and a clinical expert in the gastrointestinal tract. Draft questions were refined on the basis of feedback from adults with CP. Eligibility criteria for each objective were outlined using the relevant framework, as applicable for each of the study objectives: CoCoPop (Condition, Context, Population), PEO (Population, Exposure, Outcome), PICO (Population, Instrument, Construct, Outcome), and PICO (Population, Intervention, Comparator, Outcome). A detailed description of each objective and eligibility criteria is provided in Appendix S2. The disorders of interest were GERD, constipation, dysphagia, fecal incontinence, hepatic diseases, dental/oral cavity disorders, and colorectal cancers, according to any definition provided by study authors.

For dysphagia, objectively measured (clinical or through imaging or endoscopic methods), self‐reported swallowing problems, symptoms suggesting motor abnormalities with swallowing such as coughing or choking with food (solid or liquid), or studies reporting dysphagia by relevant International Classification of Diseases, Ninth or Tenth Revision (ICD‐9 or ICD‐10) codes were all included. Studies solely describing the Eating and Drinking Ability Classification System among adults with CP were not included as an indicator for dysphagia prevalence, as this system describes safety and efficiency of eating and drinking rather than dysphagia.^18^

Gastrointestinal tract disorders of interest and hepatic diseases differed across objectives, depending on the clinical relevance of each to the specific objective. Studies published since 1990 in any language were included. Conference abstracts, guidelines, editorials, commentaries and opinion pieces, protocols, narrative reviews, case studies, case reports, and other study designs reporting data on fewer than five people with CP were excluded. Systematic reviews that directly addressed our question of interest and that had been conducted in the previous 3 years were eligible for inclusion.

#### Population

For all objectives, the population was defined as adults with CP. Where studies included people aged 16 years and 17 years, they were included only if the study also included adults aged 18 years and older; however, no studies meeting this criterion were identified for this review. Where studies included mixed populations (i.e. both children and adults, or adults with CP and other conditions), they were included only if data on adults with CP could be extracted.

#### Additional criteria by objective

##### Objective 1

The condition was incidence or prevalence of GERD, constipation, dysphagia, fecal incontinence, hepatic diseases, dental/oral cavity disorders, and colorectal cancers. The context was any country worldwide and any setting (e.g. population‐based or hospital‐based). Cohort and cross‐sectional studies were included. All intervention study designs were excluded (e.g. randomized controlled trials [RCTs], quasi‐experimental) for this objective.

##### Objective 2

Exposures were Gross Motor Function Classification System (GMFCS) level or ambulatory status, intellectual disability, CP subtype, and obesity, assessed at any time during childhood or adulthood. The outcome was prevalent or incident GERD, constipation, dysphagia/swallowing impairments, fecal incontinence, hepatic diseases, dental/oral cavity disorders, and colorectal cancer assessed in adulthood. Cohort, case–control, and cross‐sectional studies were included. All intervention study designs were excluded (e.g. RCT, quasi‐experimental) for this objective.

##### Objective 3

All studies describing validity, reliability, responsiveness, or feasibility of tools to assess GERD, constipation, dysphagia, fecal incontinence, hepatic disease, dental/oral cavity disorders, and colorectal cancer (including screening for this cancer type) in adults with CP were included. Any quantitative study design was included. Studies that only used the instrument to assess the outcome were excluded. Studies that duplicated validation data of an instrument in a previous study (did not present new measurement property data), and studies that aimed to translate and validate an instrument in a language other than English, were also excluded.

##### Objective 4

Studies examining the effect of any intervention to treat GERD, constipation, dysphagia/swallowing impairments, fecal incontinence, hepatic diseases, dental/oral cavity disorders, or colorectal cancer in adults with CP were included. Interventions could include, but were not limited to, pharmacological interventions, surgical interventions, and therapeutic interventions. The comparator was usual care, no intervention, a modified version of the intervention, a different intervention, or placebo. Outcomes were health‐related quality of life, progression or changes in symptoms of the disease or disorder, and adverse events. RCTs, controlled before‐and‐after studies, uncontrolled before‐and‐after studies, and interrupted time series were included.

##### Objective 5

Studies examining the effect of any screening intervention to identify hepatic diseases or colorectal cancer in adults with CP were included. The comparator was usual care, no intervention, a modified version of the intervention, a different intervention, or placebo. Outcomes were incident hepatic diseases, colorectal cancer, progression of any hepatic diseases, health‐related quality of life, and adverse events. RCTs, cohort studies, controlled before‐and‐after studies, uncontrolled before‐and‐after studies, and interrupted time series were included.

### Study selection process

Two independent reviewers screened titles and abstracts, with a third reviewer resolving discrepancies between the reviewers. Separate screening checklists were developed for title/abstract screening and full‐text screening, and were piloted before use.

### Data extraction

A single reviewer extracted items for included studies using standardized data extraction forms that were piloted before use. A second reviewer verified data, and any discrepancies were discussed and the correct extraction confirmed. Separate data extraction forms were used for (1) descriptive data that were extracted for all studies (as listed in the next paragraph) and (2) data extracted for each objective. This allowed data from one study to be extracted across multiple forms, which facilitated synthesis by objective rather than an individual study.

For all studies, data on study characteristics (i.e. study design, country or countries, year[s] of data collection), source population, eligibility criteria, study setting, data collection methods, and the following participants' characteristics were extracted: age, sex, CP subtype, GMFCS level, and presence of intellectual disability. Where classification systems were not used to assess function, related data describing function such as ambulatory status or use of tube feedings (e.g. percutaneous gastrostomies) were extracted.

To address objectives 1 and 2, data describing the prevalence and incidence of gastrointestinal tract disorders or hepatic diseases for adults with CP, using raw data for denominators and numerators where available, were extracted. If available, data on the prevalence or incidence of gastrointestinal tract disorders or hepatic diseases in adults without CP or the general population, and ratios comparing the incidence or prevalence between adults with CP and adult without CP or the general population, with associated confidence intervals and *p*‐values, were extracted. Data about GMFCS level, intellectual disability, and obesity, how they were assessed, and follow‐up or study duration were extracted. Data on associations between these factors and gastrointestinal tract disorders, such as risk ratio, odds ratio, risk difference, correlation coefficients, with associated confidence intervals (CI) and *p*‐values, were extracted. Adjusted estimates were extracted. Unadjusted estimates were extracted only when no other data were available.

When examining psychometric properties of screening tools (objective 3), data were abstracted on the instrument(s) that included specific information about the subscales (but only if parts of a larger instrument were used), the construct assessed, the mode of administration, validity, reliability, responsiveness, and feasibility.

When examining effectiveness (objectives 4 and 5), data on the interventions, comparators, outcomes, adverse events, time‐points for assessments and effect estimates with 95% CI and *p*‐values (e.g. mean difference or odds ratios) were extracted. If CIs were not reported, exact *p*‐values were described. Short‐term effect was defined as 0 to 3 months post‐intervention, medium‐term as 3 to 6 months post‐intervention, and long‐term as more than 6 months post‐intervention.

### Quality appraisal

Quality appraisal was conducted independently by two reviewers using a JBI critical appraisal checklist or the COSMIN Risk of Bias checklist; conflicts were resolved through discussion. Specifically, the following JBI critical appraisal checklists were used for each study design: prevalence studies checklist for studies reporting prevalence or incidence; cohort studies checklist for cohort studies examining the association between prognostic factors and gastrointestinal tract disorders or hepatic diseases; cross‐sectional studies checklist for cross‐sectional studies reporting associations between prognostic factors and gastrointestinal tract disorders or hepatic diseases, and for studies comparing prevalence between adults with and without CP; RCT checklist for RCTs examining the effect of an intervention; quasi‐experimental studies checklist for quasi‐experimental studies examining the effect of an intervention. The COSMIN Risk of Bias checklist was used to appraise studies reporting validity of instruments to assess each of the disorders of interest. Studies were assigned an overall rating of high, low, or unclear risk of bias by taking the lowest rating of any question (i.e. ‘the worst score counts’ principle). For example, for the COSMIN checklist, if one item was rated as ‘inadequate’, the overall methodological quality of that study was rated as high risk of bias.

### Certainty in the findings

The GRADE approach was used to assess the certainty of evidence (see Appendix S3).

### Involvement of people with lived experience

Adults with CP identified the topic of this review, and review questions were refined through discussion with adults with CP. Adults with CP were involved in conducting and writing the review.

### Synthesis

A descriptive synthesis of the evidence was conducted. Summaries of the volume of information gleaned and all included studies are presented in Tables 1, 2 to 3, Tables S1 and S2, and the text. Data were synthesized by gastrointestinal tract/hepatic disorder and objective. Where single studies contributed data to more than one objective, findings were grouped according to outcomes in the text and in the tables. Detailed summaries of findings tables, with study results including effect estimates and 95% CI and *p*‐values, if reported, are provided. GRADE tables are provided with a summary of results of each comparison and confidence in the evidence.

**TABLE 1:** Summary of findings: comparison of prevalence of gastroenterological disorders or hepatic disease between adults with and without cerebral palsy.

Study	Comparison group and assessment of disorders	Gastroesophageal reflux disease	Constipation	Dysphagia	Hepatic disease
Fortuna et al.^34^	Compared with general population data from National Health and Nutrition Examination Survey (NHANES), from 2009 to 2010. Prevalence in adults with CP followed at 35 university primary care practices was assessed by medical record review, using the Rochester Health Status survey.	Not reported	Prevalence of constipation lower in adults with CP than general population for all age groups: 18–29 years, 3.6% vs 17.1%; 30–39 years, 4.9% vs 23.9%; 40–49 years, 2.0% vs 22.5%; 50–59 years, 7.3% vs 77.5%; >59 years, 5.0% vs 74.8% (*p* < 0.001 for all comparisons)	Not reported	Not reported
Henderson et al.^35^	Compared residents with and without CP. Reviewed records of group home (government or private not‐for‐profit) residents in two regions of New York State. Using the Rochester Health Status survey, nursing staff, or service coordinators reviewed medical records and recorded information about diagnoses.	Prevalence higher in adults with CP than those without CP (12% vs 9%; *p* = 0.042)	Not reported	Prevalence higher in adults with CP than in those without CP (6% vs 1%; *p* = 0.0001)	Not reported
Morad et al.^36^	Retrospective record review of medical notes of residents of residential care using Rochester Health Status survey and records from Merrick 2004. Compared residents with and without CP.	Not reported	Prevalence of constipation higher in adults with CP than in adults without CP (effect estimate not reported; *p* = 0.004)	Not reported	Not reported
Whitney et al.^44^	Adults 18–64 years and continuous enrolment in 2016 with at least one service claim in the 2016 Optum Clinformatics Data Mart Database, a US nationwide de‐identified single private‐payer administrative claims database, with and without a diagnosis code of CP were compared.	Not reported	Not reported	Not reported	Prevalence higher in adults with CP (4.1% vs 3.0%, *p* < 0.0001). Adjusted odds ratio 1.43 (95% CI 1.23–1.66; adjusted for age and sex)

Abbreviations: CI, confidence interval; CP, cerebral palsy.

## RESULTS

Study selection is described in Figure S1. Searches of PubMed, Embase, Cochrane CENTRAL, Cochrane Database of Systematic Reviews, CINAHL, and PsycINFO up to 30th September 2024 identified 3733 items for further review. Fifty additional references were identified from manual searching of reference lists of related systematic reviews and other references. After removal of duplicates, there were 1985 records. Of these, 1630 were excluded after title and abstract screening and 355 full texts were obtained. A further 323 records were excluded after full‐text screening, resulting in 32 reports for extraction. Characteristics of the included studies are described in Table S1. We did not identify any recent systematic reviews to include in our own review, as none met the inclusion criteria outlined for this type of study.

On the basis of the methods, sample size, and prevalence estimates, we assumed that three studies reported prevalence on the same sample,^19^, ^20^, ^21^ and two studies reported the association between prognostic factors and prevalence on the same sample.^19^, ^20^, ^21^ For these studies, we include data from all studies in tables, but only count data from one when describing results in text.^19^ Thus, data from 30 unique samples are reported.

Appraisals of study quality are provided in Tables [Link], [Link], [Link], [Link] to S7. All studies reporting prevalence were at high risk of bias. All studies comparing prevalence between adults with and without CP were at high risk of bias. All studies reporting prognostic factors were at high risk of bias. All studies evaluating effectiveness of interventions were at high risk of bias.

No studies reported incidence of the relevant disorders and there were no studies describing the psychometric properties of any tools used.

### Objective 1: prevalence of all gastrointestinal tract disorders and hepatic diseases

Twenty‐five studies described the prevalence of the relevant gastrointestinal tract disorders and overall hepatic diseases in unique samples of adults with CP (Table S2). Sample sizes ranged from 17 to 16 818 people.^22^, ^23^ Of these, 14 reported data on adults with CP living in the USA. Mean age ranged from 29 to 54 years.^24^, ^25^ One study included females exclusively,^26^ while for the remainder of the studies the percentage of females in each sample ranged from 36% to 74.2%, if reported.^24^, ^27^ Nine studies reported participants' GMFCS levels, and included people across all GMFCS levels.^22^, ^24^, ^27^, ^28^, ^29^, ^30^, ^31^, ^32^, ^33^

Four studies compared prevalence of gastrointestinal tract disorders or aggregated hepatic diseases between adults with and without CP (Table 1).^34^, ^35^, ^36^, ^37^ Of note, studies including comparisons generally evaluated these adults with CP and those in the same setting, for example compared with other residents in facilities or other patients in an outpatient setting. Certainty in the evidence for prevalence of all disorders and for the comparison of prevalence between adults with and without CP was very low to low (Tables S8 and S9).

#### Prevalence of GERD

Seven studies reported the prevalence of GERD, ranging from 3.3% to 42.2%.^24,26,32,34,35,38,39^ The lowest prevalence of 3.3% was found among 90 adults with intellectual disability living in a residential setting in Japan^38^ while the highest prevalence of 42.2% was found among 229 US adults identified through review of primary care practice records.^34^ Two studies reported GERD prevalence by decade of life.^32^, ^34^ Data reported on 229 US adults from primary care practices identified a GERD prevalence of 41% among 18‐ to 29‐year‐olds, 34% in 30‐ to 39‐year‐olds, 41% in 40‐ to 49‐year‐olds, 39% in 50‐ to 59‐year‐olds, and 58% among those older than 59 years.^34^ In a study of 154 adults identified from outpatient clinics, rehabilitation departments, hospitals, or organizations for disabled adults in the Republic of Korea, prevalence was 12% among 20‐ to 29‐year‐olds, 3% among 30‐ to 39‐year‐olds, 14% among 40‐ to 49‐year‐olds, and 10% among those older than 50 years.^32^

Comparing those with and without CP, the prevalence of GERD was higher among 177 adults with CP living in group homes in New York State than among adults without CP, living in the same group homes (12% vs 9%, *p* = 0.042).^35^

#### Prevalence of constipation

Seven studies reported prevalence of constipation which ranged from 4.4% to 66.7% across the studies.^25^, ^28^, ^30^, ^31^, ^34^, ^39^, ^40^ Prevalence was lowest in a US medical record review study of 229 adults followed in upstate New York primary care practices that reported constipation prevalence by decade of life: 4% among 18‐ to 29‐year‐olds, 5% in 30‐ to 39‐year‐olds, 2% in 40‐ to 49‐year‐olds, 7% in 50‐ to 59‐year‐olds, and 5% among those older than 59 years.^34^ When using a US administrative claims database to identify adults with constipation, prevalence was 10% among 8077 adults.^25^ In contrast, 65% of 91 adults recruited for a study through a US rehabilitation center were found to meet criteria for chronic constipation using the Rome III criteria, a scale developed for the general population to identify those with chronic idiopathic constipation.^31^

Prevalence of constipation among adults in upstate New York primary care practices was lower in each age group compared with US national survey data (*p* < 0.001 for all comparisons).^34^ Findings from a residential facility in Israel differed, however, in that constipation prevalence among those adults with CP was significantly higher than in residents without CP (*p* = 0.004), although the specific prevalence rate was not provided.^36^

#### Prevalence of dysphagia

Twelve studies describing unique populations reported prevalence of dysphagia ranging from 6% to 76.8%.^20^, ^21^, ^22^, ^24^, ^29^, ^30^, ^33^, ^35^, ^37^, ^41^ Prevalence was lowest, at 6%, for 177 adults living in group homes in New York State whose diagnoses were through medical record reviews.^35^ Using a sample of adults identified from a register in western Sweden, however, 29% of 153 adults were reported to have dysphagia^30^ and prevalence of dysphagia symptoms was even greater in a study of 117 adults with CP attending community centres in the Republic of Korea, where approximately 77% of adults reported that they choked on food, with a rating frequency of ‘sometimes or more’.^33^ Self‐reported frequency of drooling, as a subcomponent of prevalence ratings for oral dysphagia symptoms on a swallowing quality of life scale, was reported to be present at least sometimes, often, or almost always by 45.3% of the participants in this study.^33^

In another study of 17 adults in the Republic of Korea with dyskinetic CP and cervical dystonia, the latter diagnosis present for greater than a year, 59% of adults had aspiration or penetration of contrast in the pharynx on video fluoroscopic swallow studies.^22^ Evidence of dysphagia was much lower in this group on clinical assessment; only 17.7% had a reflexive cough with clinical swallowing testing performed with 3 mL of water.^22^

In one study comparing prevalence of dysphagia among all adults living in a group home setting in New York State, adults with CP were found to have a greater prevalence of dysphagia than those without CP (6% vs 1%, *p* = 0.0001).^35^

#### Prevalence of fecal incontinence

In three studies, prevalence of fecal incontinence was reported.^28^, ^29^, ^30^ Prevalence was 6% among adults identified through a rehabilitation center in the Netherlands, and up to 29% in a study of US outpatients with CP, who reported they rarely or never had control of formed stool.^29^, ^31^ In the last study, 31% indicated also that they rarely or never had control of liquid stool, using a standardized questionnaire developed for the general adult population.^31^, ^42^

No study compared the prevalence of fecal incontinence in those with CP and a group of adults without it.

#### Prevalence of hepatic disease

Seven studies reported prevalence of any hepatic disease or disorders, ranging from 1% to 6.3% across all studies.^21^, ^23^, ^37^, ^38^, ^43^, ^44^, ^45^ Prevalence by decade of age was similar across age groups: it was 3% in 18‐ to 30‐year‐olds, 5% in 31‐ to 40‐year‐olds and 41‐ to 50‐year‐olds, and 6% in 51‐ to 60‐year‐olds, 61‐ to 70‐year‐olds, 71‐ to 80‐year‐olds, and over‐80‐year‐olds.^45^ All studies reported hepatic diseases or prevalence in aggregate, described as ‘liver disease’, most by grouping of ICD‐9 or ICD‐10 codes used for diseases of the liver. Prevalence was summarized for codes that included (but not limited to) the following hepatic diseases: chronic viral and nonviral hepatitis, alcohol‐related diseases, metabolic dysfunction‐associated steatotic liver disease, and drug‐induced liver injury.^21^, ^23^, ^37^, ^43^, ^44^, ^45^ Thus, the term ‘hepatic disease’ has been used throughout this review. When the prevalence of hepatic disease was evaluated along with selected concurrent ICD‐10 diagnoses, the highest prevalence was noted in those with a co‐occurring chronic kidney disease.^23^

Prevalence of hepatic disease was higher in adults with CP than in adults without CP through an assessment of those enrolled in a US administrative claims database (4.1% vs 3.0%, *p* < 0.0001).^44^ After adjustment for age and sex, adults with CP were 1.43 times more likely to have any hepatic disease (95% CI 1.23–1.66) than those without CP.

#### Prevalence of dental/oral cavity disorder

Prevalence of dental/oral cavity disorders was described in five studies; this prevalence ranged from 25% to 53.4%.^24^, ^26^, ^39^, ^46^, ^47^ Dental/oral cavity disorders were reported in 26.7% of 101 US adults living in the community^46^ compared with 53.4% among 365 adults living in a residential setting in France.^39^ A study reporting on sample dental examinations performed clinically on people with CP followed at a specialized dental clinic in California, USA, documented that 25% of the population (323 people older than 20 years) had at least one missing tooth owing to trauma, periodontal disease, or extractions.^47^ The prevalence of at least one absent tooth was 59% for those older than 56 years, which was significantly greater (*p* < 0.0001) than two separate younger adult age groups: ages 21 to 35 years (prevalence 17.6%) and ages 36 to 55 years (prevalence 32.4%).^47^ The prevalence of partial or complete edentulism was 1.6% for those aged 21 to 35 years, rising to 13.0% among adults aged 36 to 55 years.^47^

### Objective 2: factors associated with gastrointestinal tract disorders or hepatic disease

Seven studies reported the association between characteristics of the people with CP and specific gastrointestinal tract disorders or hepatic disease (Table 2). Two studies included information about CP characteristics and GERD, constipation, or hepatic disease, three studies described dysphagia/swallowing impairments and their relationship to certain CP characteristics, and one study was found for fecal incontinence.^22^, ^23^, ^26^, ^30^, ^31^, ^34^, ^45^ No studies compared dental/oral cavity findings with the potential predisposing factors such as gross motor function or CP subtype.

**TABLE 2:** Summary of findings: associations between GMFCS level, intellectual disability, obesity, and prevalent gastrointestinal among adults with cerebral palsy.

Disorder	Prognostic factor	Assessment of prognostic factor	Results	*n*	Study
GERD	GMFCS level or ambulatory status	Nurses extracted data from medical records using Rochester Health Status Survey. Walking dichotomized: independent/mostly independent vs non‐ambulatory/some ambulation with assistance.	GERD prevalence in adults who walk independently/mostly independently was 38.1%; GERD prevalence was lower in adults who walk independently compared with those who are non‐ambulatory (adjusted odds ratio: 0.3; ‐95% CI 0.2–0.5, *p* < 0.001).	229	Fortuna et al.^34^
	Intellectual disability	Intellectual disability was self‐reported. It was missing for five participants.	GERD was significantly associated with intellectual disability (*p* = 0.04; no effect estimate reported).	58	Turk et al.^26^
Constipation	GMFCS level or ambulatory status	Multi‐professional team with extensive clinical experience in caring for patients with CP performed assessments and medical interviews. Medical records were reviewed by authors.	Constipation prevalence in GMFCS levels: I, 3%; II, 22%; III, 28%; IV, 42%; V, 82% (*p* < 0.001)	153	Jonsson et al.^30^
		Research assistant interviewed participants using validated questionnaire to rate GMFCS level and confirmed with records.	Constipation prevalence in GMFCS levels: I–III, 32%; IV–V, 68% (*p* = 0.18)	91	Marciniak et al.^31^
	Cerebral palsy subtype	Multi‐professional team with extensive clinical experience in caring for patients with CP performed assessments with medical interviews and medical records were reviewed by authors. Cerebral palsy subtype was classified according to the Surveillance of CP in Europe.	Constipation prevalence in CP subtype: unilateral, 3%; bilateral, 26%; dyskinetic, 62%; ataxic, 83% (*p* < 0.001)	153	Jonsson et al.^30^
	Intellectual disability	Multi‐professional team with experience in caring for those with CP interviewed and examined participant (or proxy if required) with specific authors performing medical record review. Intellectual disability was defined as IQ < 70 with deficits in adaptive skills.	Constipation prevalence with intellectual disability by IQ: IQ < 70, 68%; IQ > 70, 13% (*p* < 0.001)	153	Jonsson et al.^30^
	Obesity (BMI)	Weight (kg) and height (m) was recorded from medical record and BMI was calculated. BMI categorized as normal or underweight, overweight or obese. Obesity defined as BMI ≥ 30 kg/m^2^.	Constipation prevalence in BMI categories: normal/underweight, 56%; overweight, 20%; obese, 24% (*p* = 0.91)	91	Marciniak et al.^31^
Dysphagia	GMFCS level or ambulatory status	Multi‐professional team with experience caring for people with CP interviewed and examined participant (or interviewed proxy if required).	Dysphagia prevalence by GMFCS level: I, 8%; II, 25%; III, 33%; IV, 46%; V, 82% (*p* < 0.001)	153	Jonsson et al.^30^
		Physiatrist conducted assessment for determination of GMFCS level. Video fluoroscopic findings were evaluated using the video fluoroscopic dysphagia scale.	Total score on video fluoroscopic dysphagia scale was not associated with GMFCS level (rho = 0.212, *p* = 0.414)	17	Seo et al.^22^
	Cerebral palsy subtype	Multi‐professional team with experience caring for those with CP interviewed and examined participant (or interviewed proxy if required) and specific authors performing medical record review.	Dysphagia prevalence in CP subtype: unilateral, 13%; bilateral, 36%; dyskinetic, 55%; ataxic, 17% (*p* < 0.001)	153	Jonsson et al.^30^
	Intellectual disability	Multi‐professional team with experience caring for those with CP interviewed and examined participant (or interviewed proxy if required), with specific authors performing medical record review. Intellectual disability defined as IQ < 70 with deficits in adaptive skills.	Dysphagia prevalence in those with intellectual disability: IQ < 70, 62%; IQ > 70, 20% (*p* < 0.001)	153	Jonsson et al.^30^
		Medical conditions were identified by searching for specific ICD‐10‐CM codes that are attached to individual claims; 20% random sample of the Medicare fee‐for‐service administrative claims data source used.	Dysphagia prevalence in CP only, 13.5%; CP and intellectual disability, 24.3%; CP and epilepsy and intellectual disability, 34.8% (effect estimate and *p*‐value not provided)	16 728	Whitney and Basu^19^
		Medical conditions were identified by searching for specific ICD‐10‐CM codes that are attached to individual claims; 20% random sample of the Medicare fee‐for‐service administrative claims data source used.	Dysphagia prevalence in CP only, 13.5%; CP and intellectual disability, 24.3%; CP and epilepsy and intellectual disability, 34.8% (effect estimate and *p*‐value not provided)	16 728	Whitney et al.^20^
Fecal Incontinence	GMFCS level or ambulatory status	Research assistant interviewed participants using validated questionnaire to rate GMFCS level and confirmed with records.	Prevalence of reporting ‘rarely or never able to control liquid stool’ versus GMFCS level: I–III, 9%; IV–V, 44% (*p* = 0.0004) Prevalence of reporting ‘rarely or never able to control accidental loss solid stool’ in GMFCS level: I–III, 12%; IV–V, 39% (*p* = 0.008)	91	Marciniak et al.^31^
	Obesity (BMI)	Weight (kg) and height (m) was recorded from medical record and BMI (kg/m^2^) was calculated. BMI categorized as normal or underweight, overweight or obese. Obesity was defined as having a BMI ≥30 kg/m^2^.	Prevalence of reporting ‘rarely or never able to control liquid stool’ in BMI category: normal/underweight, 38%; overweight, 13%; obese, 24% (*p* = 0.16) Prevalence of reporting ‘rarely or never able to control accidental loss solid stool’ in BMI category: normal/underweight, 33%; overweight, 6%; obese 29% (*p* = 0.10)	91	Marciniak et al.^31^
Hepatic disease	Intellectual disability	Medical conditions were identified by searching for specific ICD‐10‐CM codes that were attached to individual claims; a 20% random sample of the Medicare fee‐for‐service administrative claims data source was used.	Mild to severe liver disease prevalence in those with CP only, 4.7%; CP and intellectual disability, 4.8%; CP and epilepsy and intellectual disability, 4.9% (effect estimate and *p*‐value not provided)	16 728	Whitney and Basu^19^
		Medical conditions were identified by searching for specific ICD‐10‐CM codes that are attached to individual claims; 20% random sample of the Medicare fee‐for‐service administrative claims data source used.	Mild to severe liver disease prevalence: CP only, 4.7%; CP and intellectual disability, 4.8%; CP and epilepsy and intellectual disability, 4.9% (effect estimate and *p*‐value not provided)	16 728	Whitney et al.^20^
		Medical conditions were identified by searching for specific ICD‐10‐CM codes that are attached to individual claims; 20% random sample of the Medicare fee‐for‐service administrative claims data source used.	Age‐adjusted odds ratio of liver disease among adults with CP with co‐occurring intellectual disability compared with those without intellectual disability: odds ratio 1.01 (95% CI 0.87–1.17)	16 818	Whitney et al.^45^

Abbreviations: BMI, body mass index; CI, confidence interval; CP, cerebral palsy; GERD, gastroesophageal reflux disease; GMFCS, Gross Motor Function Classification System; ICD‐10‐CM, International Classification of Diseases, 10th Revision, Clinical Modification.

Sample sizes ranged from 17 to 16 818 people.^22^, ^45^ Of these seven studies, five reported data on adults with CP living in the USA. Certainty in the evidence for all prognostic factors for these disorders was very low, except for associations between intellectual disability, and dysphagia and hepatic disease, where there was moderate certainty in the evidence (Tables [Link], [Link], [Link], [Link]).

#### GERD and characteristic prognostic factors

The prevalence of GERD was lower in US adults who walked independently than in those who were non‐ambulatory (adjusted odds ratio 0.3, 95% CI 0.2–0.5, *p* < 0.001),^34^ as documented upon chart review of people followed in university‐affiliated primary care practices in upstate New York. The prevalence of GERD was also associated with intellectual disability in a study of US females (*p* = 0.04), but the direction and strength of association was not reported.^26^

#### Constipation and characteristic prognostic factors

A study of 91 adults recruited at one rehabilitation department in the USA reported no difference in prevalence of constipation, whether a participant's gross motor mobility was among GMFCS levels I to III compared with IV to V.^31^ However, a second study of 153 adults identified from a register in western Sweden reported that the prevalence of constipation increased with increasing GMFCS level, from 3% for those in level I to 82% in level V (*p* < 0.001). In that study, constipation was most prevalent among adults with ataxic CP (83%), followed by dyskinetic (62%), spastic bilateral (26%), and spastic unilateral (3%) CP (*p* < 0.001).^30^ Prevalence of constipation was also higher in adults with intellectual disability than in adults without intellectual disability (68% vs 13%, *p* < 0.001).^30^ Prevalence of constipation did not differ across body mass index categories (*p* = 0.91).^31^

#### Fecal incontinence and characteristic prognostic factors

Fecal incontinence was associated with GMFCS level: a higher prevalence was found in adults classified in GMFCS levels IV or V (reporting they rarely or never were able to control liquid stool) than in individuals in GMFCS levels I to III (44% vs 9%, *p* = 0.0004).^31^ Similarly, more adults in GMFCS levels IV or V reported rarely or never being able to control accidental loss of solid stool compared with those in GMFCS levels I to III (39% vs 12%, *p* = 0.008).^31^

#### Hepatic disease and characteristic prognostic factors

Prevalence of a hepatic disease according to intellectual disability status in a US population of adults enrolled in an administrative claims database was reported to be 4.7%, 4.8%, and 4.9%^20^ when evaluating those with CP only, with CP and intellectual disability, and with CP, intellectual disability, and epilepsy respectively. No statistical test was used to compare prevalence, however. A study of 16 818 adults enrolled in an administrative claims database reported no association between prevalence of hepatic disease and intellectual disability (age‐adjusted odds ratio 1.01, 95% CI 0.87–1.17).^45^

#### Dysphagia and characteristic prognostic factors

Total score on a video fluoroscopic dysphagia scale was not associated with GMFCS level among 17 Korean adults with dyskinetic CP who had cervical dystonia for more than a year (rho = 0.212, *p* = 0.414),^22^ which contrasts with a study of 153 adults identified from a register in western Sweden which found prevalence of dysphagia was higher as GMFCS level increased, from 8% in those in GMFCS level I to 82% in those in GMFCS level V (*p* < 0.001).^30^ In the same study, intellectual disability was associated with prevalence of dysphagia (62% with intellectual disability vs 20% without intellectual disability, *p* < 0.001).^30^ Jonsson et al. noted as well that dysphagia was most prevalent in adults with dyskinetic CP (55%), followed by bilateral spastic CP (36%), ataxic CP (17%), and unilateral spastic CP (13%) (*p* < 0.001).^30^

Using US administrative claims data, 16 728 adults with CP were found to have dysphagia, as defined by relevant ICD‐10 codes. The prevalence of dysphagia ranged from 14% to 35% in adults with CP, depending on associated co‐occurring condition. In adults with CP only, the prevalence was 14%, compared with 24% in adults with CP and co‐occurring intellectual disability, while prevalence was 35% in adults with CP, intellectual disability, and epilepsy.^20^ No formal statistical test was conducted to compare prevalence across these groups.

No studies were identified that compared prevalence of dental/oral cavity disorders in adults with CP and those without CP, or evaluated the prevalence in association with other CP characteristics or co‐occurring medical conditions.

### Objective 3: evidence for screening tools to assess disorders among adults with CP

No studies assessing the psychometric properties of screening tools for gastrointestinal tract disorders (including colorectal cancer) or for hepatic disease were identified.

### Objective 4: effectiveness of interventions for gastrointestinal tract disorders or hepatic disease

We identified four studies assessing intervention effectiveness; these studies' details can be found in Table 3. The studies included in total 103 adults with CP and described interventions for dysphagia (two studies) or dental/oral cavity disorders (two studies). Two were RCTs,^48^, ^49^ while two were uncontrolled before‐and‐after studies.^50^, ^51^ Certainty in the evidence for all comparisons was very low (Tables S14 and S15). Sample size ranged from 10 to 62 participants.^50^, ^51^ Studies were conducted in Sweden, France, Denmark, and Portugal. We did not find any studies assessing the effectiveness of interventions to treat GERD, constipation, fecal incontinence, or hepatic disease in adults with CP.

**TABLE 3:** Summary of evidence: interventions for gastroenterological disorders or hepatic disease.

Study	Design	*n*	Intervention	Comparator	Outcomes	Effect	Adverse events
Bizarra and Ribeiro Graca^49^	Randomized controlled trial	62	**Education and training intervention** Caregivers and participants in the intervention group facilities received information about oral health and disease by a faculty member of a university dental hygienist program. There was also a demonstration of toothbrushing and adaptive techniques. Return demonstration of techniques performed by the caregivers. Individual oral hygiene monitoring was also performed five times, over first 2 months postintervention for reinforcement.	**Usual care** Toothbrushing twice a day.	**Clinical examination indices** ‐ Gingival Index. ‐ Simplified Oral Hygiene Index with subcomponent debris index. Assessed at baseline, 2 months and 6 months post‐intervention.	**Simplified Oral Hygiene Index** Intervention group vs control group 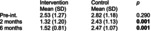 **Gingival Index** Intervention group vs control group 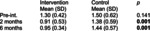 Comparing examination findings for adult participants with cerebral palsy at the facilities where the educational intervention was performed with participants at the usual care facilities, there were statistically significant differences in means for both oral health indices (Oral Hygiene Index and gastrointestinal tract) at 2‐month and 6‐month follow‐up.	None reported
Cahlin et al.^48^	Randomized controlled trial	12	**Botulinum neurotoxin type A (Botox)** 100 units in 1 mL normal saline or equivalent volume of isotonic saline, was injected bilaterally in the masseters (30 units/muscle) and temporalis muscles (20 units/muscle). Injections performed with electromyographic guidance.	**Saline injections** Equivalent volume saline was injected into the same muscle groups of the control participants.	**‐ Bite force** **‐ Chewing efficiency** **‐ General Oral Health Index (Swedish Version)** **‐ Questionnaire** (Prevalence of bruxism, pain in jaws, ability to chew, and ability to talk during preceding week.) Assessed at short term (2 weeks), intermediate (16 weeks), post‐injection.	No between‐group differences for any of the assessed outcomes (*p* > 0.05).	None reported
Davout et al.^50^	Uncontrolled before‐and‐after study	2	Percutaneous gastrostomy placement.	**No comparato**r.	**Bodyweight** ‐ Bodyweight increase at least 15%*: n* (%) ‐ Weight gain (kg) Median weight change baseline to long‐term follow‐up Assessed at long‐term follow‐up	15 (88.2%) gained at least 15% bodyweight. Baseline to follow‐up: median change 4.2 kg (interquartile range 1.0–8.8; ***p* < 0.01**)	**Procedure‐related complications** Short term (up to 72 hours): ‐ Fever: *n* = 6 (31.6%) ‐ Localized wound infection with fever: *n* = 3 (50%) ‐ Transient hypoxia: *n* = 7 (36.8%) ‐ Acute respiratory failure: *n* = 1 (5.2%) ‐ Transient ileus: *n* = 2 (10.5%) Intermediate (>72 hours) to long‐term follow‐up: ‐ Non‐severe stomal incidents: *n* = 9 (52.9%) ‐ Mobility decline due to feeding tubes/wound management during mobilization: *n* = 4 (25%) Long term, percutaneous endoscopic gastrostomy‐related: ‐ Oral feedings no longer used in *n* = 5 (26%) who were fully or partly orally fed before procedure.
Pingel et al.^51^	Uncontrolled before‐and‐after study	10	**Singing lessons**, 1‐hour duration, twice weekly one‐on‐one sessions with professional singing teacher, individualized by participants' needs for major speech and/or swallow including exercises for motor control of lips and tongue during singing.	No comparator.	**Self‐reported difficulty with supraglottic swallow exercise** 1–10 point scale (1 = not difficult at all; 10 = very difficult)	**Self‐reported difficulty with supraglottic swallow exercise** 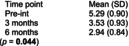	No participants reported negative side effects from singing intervention.
					**Self‐reported difficulty with normal swallowing exercise** 1–10 point scale	**Self‐reported difficulty with swallowing exercise** 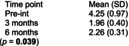	
						**Self‐reported difficulty with speaking** 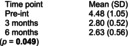	
						**Self‐reported changes in eating and drinking ability** Time point 6 months Eating ability: 80% reported positive effects on eating skills Drinking ability: 50% reported positive effects	
						**Mealtime eating and drinking abilities** 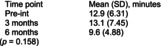	
						**Self‐reported drooling frequency** 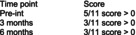 Three participants reported lower frequency on the scale at the 6‐month rating compared with the pre‐intervention rating.	

*Note*: Bold type for *p*‐values indicates statistical significance.

Abbreviations: Pre‐int., preintervention; SD, standard deviation.

Davout et al. reported complications and outcomes in a group of 26 French adults with CP who were referred for percutaneous endoscopic gastrostomy placement required for dysphagia and/or tube replacement to maintain nutrition.^50^ Percutaneous placement could not be completed in seven people, generally because of the inability to reliably distinguish stomach location using transillumination. The authors indicated severe scoliosis was the most common cause of this limitation. Short‐term (3 days or less) complications were generally respiratory and long‐term complications were most often stomal. At follow‐up after successful placement of the percutaneous endoscopic gastrostomy tube, at a median of 23 months, median weight gain was 4.2 kg (IQR 1.0–8.8; *p* < 0.01), with 88% of participants gaining at least 15% of their baseline bodyweight.^50^

Regarding dental/oral cavity disorders, one RCT evaluated the efficacy of an educational intervention provided both for caregivers performing dental/oral hygiene for adults with CP residing at one of four residential facilities or for adults with CP who were independent in this function.^49^ Interventions were randomized by facility and included education for caregivers in toothbrush techniques and head/body positioning to help with performance of oral hygiene and for residents with CP if they were independent for this function. Outcomes of gingival health status and oral hygiene were measured through examination and scoring participants on the Gingival Index and the Simplified Oral Hygiene Index. Mean ratings on the Simplified Oral Hygiene Index were significantly lower (better) at 2 months and 6 months after intervention for adults independent or requiring a caregiver for oral hygiene (mean difference not reported; *p* = 0.001 and *p* = 0.001 respectively) compared with the control group. Similarly, mean ratings on the Gingival Index were significantly lower (better) at 2 months and 6 months after intervention for adults independent or requiring a caregiver for oral hygiene (mean difference not reported; *p* = 0.001 and *p* = 0.001 respectively) compared with controls.

The second study, an RCT assessing dental/oral cavity function^48^ in those with bruxism, compared the use of botulinum neurotoxin (Botox) injections with an equivalent volume of isotonic saline distributed across relevant muscles. No significant differences were found comparing the two injection types at follow‐up, which included ratings of the ability to chew, chewing efficiency, bruxism, pain in the jaw, and the General Oral Health Assessment Index (*p* > 0.05).^48^ (Table 3).

The effectiveness of singing lessons on dysphagia and oral motor activities (speech) in adults with CP was assessed in an uncontrolled before‐and‐after trial with singing lessons performed twice a week for 6 months.^51^ Fifty per cent reported positive effects on drinking skills using a scale of 0 to 10. Eating skills (effect estimate not reported, *p* = 0.0003) and oral motor skills (effect estimate not reported, *p* = 0.0001) improved significantly over time.

### Objective 5: effectiveness of screening for hepatic disease or colorectal cancer

No studies were found addressing this objective.

## DISCUSSION

This review presents information about the prevalence, predictors, and interventions that have been described for gastrointestinal tract and hepatic‐related diseases in adults with CP. We found no information about screening and prevalence of colorectal cancer among adults with CP. Where evidence was found, the results of this review must be interpreted with caution, as certainty in the evidence relating to all objectives was low to very low. For diagnosis prevalence, in part this was due to disparate findings across studies. Understanding differences in samples and methods used in this review's studies may help to direct recommendations for improvements in care, as well as to improve the quality of future studies of gastrointestinal tract and hepatic disorders in adults with CP.

As GERD is one of the most common gastrointestinal tract disorders in the Western world, it is not surprising that this gastrointestinal tract disorder is reported as a frequent co‐occurring condition in adults with CP. GERD symptoms in the general US adult population have been reported to vary from 6% to 30%, dependent on symptom classification for frequency and duration.^52^, ^53^, ^54^ In adults with CP, a recent systematic review and meta‐analysis of four studies reported a 16% pooled prevalence of GERD.^12^ Rates reported in these studies of adults with CP seem comparable or higher to rates reported in adult populations of the same country, and a single US study directly compared GERD prevalence rates in their participants as higher in adults with CP than in those without (12% vs 9%).^35^ Most risk factors found to be associated with GERD in the general US population were not considered in the studies of adults with CP. Although abdominal obesity is one risk factor for GERD in US adults, no studies in our review examined this risk factor.^52^ The prevalence of GERD also differs when considering the country studied, with the adult populations in the USA and European countries reporting higher rates than those in East Asia.^24^, ^26^, ^32^, ^34^, ^35^, ^38^, ^39^ Across the included studies in this review, adults with CP from Japan and the Republic of Korea seemed to have lower prevalence rates, at 3.3% and 9.1% respectively, compared with the US and European samples of adults with CP we have described (prevalence 12%–42.4%).^30^, ^37^ Thus, interestingly, although overall GERD prevalence may be increased in adults with CP, our relative rates by country are consistent with the lower prevalence reported in studies from East Asian countries in the general adult population. Therefore geographical origin should be a consideration when rates and interventions are described in adults with CP.^55^

Among US studies included in this review, the lowest prevalence of GERD was 12%, which was documented through chart review for adults with CP living in group homes, 47% of whom had severe to profound intellectual disability.^35^ It is important to note that prevalence rates in this previous study, as well as others that include individuals with profound intellectual disability, have the potential to underestimate GERD as evaluation and/or treatment is generally initiated with classic symptom complaints of heartburn and effortless regurgitation in the posterior pharynx; screening is not routinely performed. Individuals with significant intellectual disability may find symptoms of GERD difficult to describe. GERD may also present with symptoms of extra‐esophageal disorders, such as asthma, cough, and sore throat, making diagnosis more challenging.^52^ Confirmation testing for GERD, such as ambulatory pH monitoring, may be difficult to perform in some individuals with CP, such as those with severe movement disorders or intellectual disability, as testing can be uncomfortable and requires full participation from the patient. We did not, however, find data specifically assessing the experience of adults with CP requiring GERD testing. Families or caregivers of those with significant intellectual disability may also be more reluctant to have testing performed because of risks associated with the confirmatory testing as well as the additional procedures that increase risk, such as anesthesia for esophagogastroduodenoscopy. Although anesthesia alone may result in a greater risk for the individual being assessed, GERD itself may lead to long‐term complications including dental/oral disorders (those above as well others such as dental sensitivity and tooth decay), esophageal strictures, erosive esophagitis, or even malignancies such as esophageal adenocarcinoma.^52^, ^56^ Alternative clinical signs of halitosis, gingivitis, and erosive tooth lesions with or without decay may be noted through clinical examinations, suggesting the presence of GERD. Future studies need to identify whether risk factors in those with CP are similar to those of the general population. Safe, best‐practice screening mechanisms, particularly for those with more difficulty communicating symptoms on history, will need to be documented. Chart review methodology alone in those with intellectual disability may result in unrecognition of the extent of the problem.

Similar to GERD, rates of constipation were disparate across the studies included in this review.^25^, ^28^, ^30^, ^31^, ^34^, ^39^, ^50^ In a previous systematic review and meta‐analysis, the pooled prevalence of constipation in adults with CP was 16.9% and similar to pooled prevalence rates for the general population (at approximately 14%).^12^, ^57^ One study in this review used a standardized diagnostic questionnaire (Rome III criteria) which was created to assess chronic idiopathic constipation in adults. In contrast to studies reporting rates by record review or ICD codes, the prevalence rate found for adults with CP in this study was much higher (65%) and, across GMFCS levels, prevalence was greater in the non‐ambulatory adults with CP.^31^ This last finding is consistent with constipation rates in US adults in whom constipation is associated with a sedentary lifestyle. A population‐based study of adults without CP that used an updated version of Rome criteria (IV) found 24% of US adults experienced chronic idiopathic constipation.^58^ Of note, 60% of the participants with chronic idiopathic constipation in the general US population in that study said that they never discussed their constipation symptoms with their healthcare provider.^58^ Furthermore, an additional study of adults with CP that identified prevalence of constipation through chart review reported bowel obstructions in their population were more prevalent than constipation, which is an unusual finding.^34^ A subsequent study using the same survey instrument, but in which evaluations were limited to individuals in group homes, reported that ‘bowel obstruction’ was often related to severe constipation.^35^ The lower prevalence of constipation among the studies using chart review methodology again suggests that this method may result in an underestimation of the true prevalence in adults with CP. Future studies should use more standardized definitions for constipation, obstipation related to fecal impaction, and obstructed defecation, such as seen with pelvic floor dysfunction. The Rome IV criteria are validated symptom‐based criteria used for diagnosing these conditions in the general adult population, and are an example of an instrument that could be considered for validation for use in adults with CP. The need for careful follow‐up by caregivers regarding constipation signs and symptoms in more vulnerable populations of adults with CP is suggested by these reports of obstruction. Future studies should identify practical methods to regularly screen for constipation symptoms in addition to evaluating interventions. Finally, assessment as to whether monitoring for constipation prevents complications is imperative. It has been noted recently that the composition of gut microbiota differs among those with constipation in the general population, a finding that could influence susceptibility to intestinal disease including colorectal cancer.^59^ Such relationships are still under study. Nonetheless, constipation (or changes in this symptom) can be a presentation of colorectal cancer, and thus ascertaining and follow‐up of constipation symptoms may have significant long‐term health consequences.

Among studies in this review, rates for difficulty swallowing were most often identified through inquiry about swallowing and choking on food, rather than imaging or examination while swallowing. A single study that reported results of video fluoroscopic swallow studies in adults with CP was limited to those with dyskinetic CP and cervical dystonia.^22^ Although results from video fluoroscopic swallow studies were not associated with GMFCS levels, they were associated with a scale that specifically measured the degree of cervical dystonia, the Toronto Western Spasmodic Torticollis Rating Scale. Total and pharyngeal scores on these studies were significantly correlated with this cervical dystonia scale rating, indicating that, for this group of adults with CP, assessments of the cervical dystonia may be more important than overall gross motor function when determining the need for further dysphagia assessments. Of note as well, the rate of aspiration of thin liquids into the pharynx on video fluoroscopic swallow studies was twice that reported in interviews with participants/caregivers for clinical aspiration symptoms. Thus, unrecognized aspiration by patients and/or their caregivers may need to be considered in adults with dyskinetic CP.^22^

Fecal incontinence was another disorder found to be highly variable in prevalence,^28^, ^29^, ^31^ and at rates greater than the general population. A recent systematic review in the general population reported a global pooled prevalence of 8.0% for fecal incontinence in adults,^60^ with a prevalence even lower if standardized (Rome I‐IV) criteria are used to identify incontinence.^60^ A pooled rate for fecal incontinence in adults with CP reported a higher prevalence, at 14.6%,^12^ and in this present review we observed the highest rates with the use a standardized validated questionnaire. Furthermore, prevalence of fecal incontinence was associated with greater gross motor limitations, as assessed by a higher GMFCS level;^31^ and one study in this review noted fecal incontinence was detrimental to the quality of life of these adults in that 15% of all participants with CP had moderate to moderate–severe interference with their quality of life owing to bowel movements.^31^ These quality of life effects are consistent with findings in the global general population.^60^ Future prospective studies should proactively query adults with CP about fecal incontinence problems, which may be valuable if identification and management improves quality of life.

Although the prevalence of hepatic disease seemed to be increased, it was only reported as an aggregate: that is, across all diagnoses of diseases related to the liver. Almost all studies identified in the review used ICD codes and claims‐based data, codes that included many diseases of the liver. Additionally, there was apparent overlap in the populations describing hepatic disease found through our systematic literature review. Adults with CP are probably at risk for hepatic disease related to at least several co‐occurring conditions. The use of antiseizure medication is increased in this population, a drug class known to cause drug‐induced liver injury. Other risk factors are malnutrition (undernutrition) and, alternatively, overnutrition with metabolic dysfunction‐associated steatotic liver disease (previously referred to as non‐alcoholic fatty liver disease), as there is an increased prevalence of cardiometabolic syndrome in this population. Circulating proinflammatory cytokines seem to be upregulated in children with CP and, although speculative, may contribute early inflammatory changes.^61^ Given the recent report of hepatic fibrosis or steatosis on liver ultrasound or transient elastography (FibroScan) in asymptomatic children with CP, rates reported using liver function on blood tests only may underestimate prevalence both in children and in adults.^62^ Future studies are urgently needed using appropriately sensitive measures to screen and confirm the presence of hepatic disease and to identify other factors contributing to this increased prevalence in adults with CP compared with the general population.

Dental/oral cavity disorders in adults with CP are complex and multifactorial. They are a significant source of morbidity in this population.^47^, ^63^ Additionally, dental/oral cavity disorders along with dysphagia have been shown to contribute to respiratory mortality in adults.^63^, ^64^

Oral health has undergone extensive study in children with CP. Among problems reported with increased prevalence are dental caries, periodontal and gingival disease, dental anomalies, and malocclusion.^65^, ^66^ The reported problems continue to adulthood, with increased loss of teeth, which is generally due to trauma, caries, and periodontal disease leading to partial and complete edentulism throughout adulthood.^47^ For those with CP, impaired limb motor function and movements can be a barrier for both children and adults in performing adequate oral hygiene. A survey of caregivers of adults with developmental disabilities found among factors interfering with oral hygiene were the adults' inability to rinse and spit, or which occurred because of aspiration during oral hygiene tasks.^49^, ^67^ Further barriers to oral hygiene may include the need for anesthesia in adults with CP for dental cleaning. A greater mean level of decayed, missing, and filled teeth has been reported for adults with CP when compared with adults with Down syndrome, mainly driven by higher levels of decayed and missing teeth.^68^ Bizarra et al. did find that, with appropriate training of care providers, oral hygiene and gingival health in adults with CP can be significantly improved; however, these findings need confirmation in future studies.^49^

Adults with CP have greater risks of oral health conditions and require strategies to facilitate oral hygiene care. Many of the selective preventive, behavioral, and treatment strategies effective for children with special needs can also be adapted for adults with CP. The American Association of Pediatric Dentistry has published management guidelines specifically for children with special needs.^69^, ^70^

We were unable to identify any studies reporting colorectal cancer prevalence, incidence, or mortality rates for adults with CP. Risk factors associated with colorectal cancer in the general population, for example lower activity levels and obesity, probably put many adults with CP at heightened risk.^71^ As circulating proinflammatory cytokines seem to be upregulated in children with CP, this could affect cancer rates.^72^ Such effects have not been clarified in adults with CP, however.

Unfortunately, although screening rates for colorectal cancer among adults with CP were not found for this present review, a study published almost two decades ago reported cancers of the digestive organs and peritoneum were twice as likely to contribute to mortality over the lifespan (ages 2 years to adulthood) for individuals with CP than for those without CP.^14^ Specifically for digestive disease cancers, standardized mortality ratios were increased in that study for esophageal, colon, and liver cancers; both children and adult mortality rates were combined, however.^14^ In contrast, a recent report using information from practices in England that combined four types of malignant neoplasm, including colorectal cancer, concluded that mortality for cancer was not increased in adults with CP for these combined cancers.^13^ Of note, studies reporting colon cancer mortality in combination with another cancer (such as lung cancer) that may have a lower prevalence in adults with CP, could overall underestimate the impact of cancer in these adults.^13^, ^14^ Similarly, reporting data including children with adults, when specific cancers such as colorectal cancer are more likely to be found with increasing age, may also undermine concerns for this disease in adulthood.^14^ These issues need to be considered when designing studies to assess cancer rates in adults with CP.

Currently, colorectal cancer screening is recommended for people at average risk in the USA beginning at age 45 years. For adults with CP, common co‐occurring conditions (constipation, dysphagia, and cognitive impairment) and limitations in the ability to perform independent toilet transfers are barriers to people agreeing to colonoscopy as a method of screening. Despite the potential benefit of colonoscopy for both for screening and treatment, the latter due to the ability to remove any isolated malignant adenomatous polyps identified during the procedure, the American College of Physicians published alternative screening methods, recognizing barriers to colonoscopy exist even in the general US population. Updated recommendations published in the 2023 guidelines from the American College of Physicians include the following in the absence of risks factors such as genetic or family history or other red flags for cancer: testing such as fecal immunochemical, high sensitivity guaiac fecal occult blood testing, or flexible sigmoidoscopy, alone or in combinations.^73^ It is worthy of note that negative stool tests need to be repeated within a shorter interval than a negative colonoscopy. In addition, stool tests do not replace colonoscopy, as colonoscopy will probably be recommended, despite the barriers, as the next follow‐up if stool testing yields a positive result. For adults with CP, all these options are probably important, and should be considered along with an individual's cancer risk factors, in order to reduce barriers to, and facilitate, colorectal cancer screening. Barriers to health screenings for other cancers have been described in those with disabilities. These barriers include cost, access to facilities or equipment, lack of healthcare provider referrals, and physical and cognitive limitations.^74^, ^75^, ^76^ Similar barriers are likely to apply for colorectal cancer screening, and deserve to be studied, along with confirmation of screening and cancer rates.

Although common in the general US population, studies highlighting biliary tract or gall bladder disorders in adults with CP and meeting inclusion for the study were not identified in our search. This topic area was not identified as a priority question for the adults with lived experience with CP who were providing input for this study.

Articles included in this review had a range of sample sizes; in particular, studies evaluating interventions represented only a small number of adults with CP. The findings about these gastrointestinal tract diseases and hepatic disorders are reported for adults with CP in relatively high‐income countries only and thus findings may not be appliable to adults with CP in low‐ and middle‐income countries. The specific gastrointestinal tract disorder(s) being reported were not always well‐defined in the studies we reviewed, nor was the length of time the condition was present in the adult with CP. Furthermore, scales for outcome assessment have not been validated for adults with CP. These factors probably contributed to the heterogeneity in prevalence rates identified in this review.

## CONCLUSIONS

Adults with CP have a higher prevalence of GERD, dysphagia, and hepatic disease than adults without CP. A lower prevalence of GERD, constipation, and fecal incontinence was identified in adults with better gross motor function, while a higher prevalence of GERD, constipation, and dysphagia was found in adults with CP and intellectual disability. Despite this increased risk, there is a lack of evidence to support screening and management of these disorders. Underdiagnosis may be more problematical in adults with CP with co‐occurring intellectual disability. Thus, for disorders where evaluation is driven by an individual reporting specific symptoms, better information about risk factors and screening approaches is needed for this population of patients. The prevalence of hepatic disorder seems higher in adults with CP than in the general US population but specific diagnoses need to be distinguished to guide screening and intervention recommendations. Evaluations of colorectal cancer screening and mortality rates have been neglected among adults with CP. Future studies should focus on better quantification of screening, incidence, and mortality rates, given the likely significant risk factors in the population of adults with CP.

There have been few assessments of interventions in adults with CP for any of the disorders described in this review. Singing as an intervention for dysphagia and oral motor function was reported to be helpful, and a novel and safe intervention. Future studies, however, will need to explore this intervention further, given the time‐intensive nature and resources required for completion of the study. Targeted education and training both of caregivers and of adults with CP also seems promising to improve dental and oral hygiene. Overall, evaluation of management strategies for these gastrointestinal tract disorders and hepatic disease is vital, especially as to whether screening and interventions lead to improvements in overall health, health‐related quality of life, and, particularly for colorectal cancer, improved mortality rates.

## FUNDING INFORMATION

This project was supported by a grant from the Cerebral Palsy Foundation.

## CONFLICT OF INTEREST STATEMENT

The authors have stated that they had no interests which might be perceived as posing a conflict or bias.

## Supporting information


**Appendix S1:** Search strategy for PubMed.

**Appendix S2:** Eligibility criteria by question.

**Appendix S3:** GRADE criteria.

**Figure S1:** PRISMA diagram.

**Table S1:** Description of included studies.

**Table S2:** Summary of findings: prevalence of gastrointestinal disorders and hepatic disease among adults with cerebral palsy.

**Table S3:** Quality appraisal of prevalence studies.

**Table S4:** Quality appraisal of studies comparing prevalence between adults with and without CP.

**Table S5:** Quality appraisal of cross‐sectional studies examining prognostic factors for GI and hepatic disorders.

**Table S6:** Quality appraisal of randomized controlled trials.

**Table S7:** Quality appraisal of quasi‐experimental studies.

**Table S8:** Summary of clinical evidence profile for prevalence studies.

**Table S9:** Summary of clinical evidence profile for studies comparing those with CP versus those without CP.

**Table S10:** Summary of clinical evidence profile for comparison: GMFCS levels I, II, III, IV, and V or ambulatory vs not ambulatory.

**Table S11:** Summary of clinical evidence profile for comparison: CP subtype.

**Table S12:** Summary of clinical evidence profile for comparison: Intellectual Disability.

**Table S13:** Summary of clinical evidence profile for comparison: Obesity.

**Table S14:** Summary of clinical evidence profile comparison: non‐pharmacological intervention compared to no intervention or usual care.

**Table S15:** Summary of clinical evidence profile comparison: pharmacological intervention compared to no intervention or usual care.

## Data Availability

No data is available beyond that published, including in the online supplementary material.
