# Supplementary material for: Gastroenterological disorders and hepatic disease in adults with cerebral palsy: A systematic review
Source: Dev Med Child Neurol. 2025 Oct 30;68(3):313–31. doi: 10.1111/dmcn.70034 (PMC12875176; doi:10.1111/dmcn.70034)
Supplement: Supplementary file 1 — Appendix S1: Search strategy for PubMed. [file DMCN-68-313-s001.docx]

**Appendix S1: PubMed Search Strategy**

| Set # | Search Strategy |
| --- | --- |
| #1 | "cerebral palsy" OR "Cerebral Palsy"[MeSH] OR "cerebral paresis"[tiab] |
| #2 | "Digestive System Diseases"[MeSH] OR "Gastrointestinal Diseases"[MeSH] OR "Myoelectric Complex, Migrating"[MeSH] OR gastroenterology[tiab] OR Gastrointestinal[tiab] OR "digestive system"[tiab] OR stomach[tiab] OR gallbladder[tiab] OR esophageal[tiab] OR esophagus[tiab] OR intestine[tiab] OR intestines[tiab] OR colon[tiab] OR rectum[tiab] OR pancreas[tiab] OR "bile duct"[tiab] OR "bile ducts"[tiab] OR liver[tiab] OR "abdominal pain"[tiab] OR Achlorhydria[tiab] OR "aveolar bone atrophy"[tiab:~0] OR "aveolar bone loss"[tiab:~0] OR "aveolar reabsorption"[tiab:~0] OR "aveolar resorption"[tiab:~0] OR "ampulla of vater"[tiab] OR "anorectal abnormalities"[tiab] OR appendicitis[tiab] OR "Barrett Esophagus"[tiab] OR "bile duct disease"[tiab] OR "bile duct diseases"[tiab] OR "bile reflux"[tiab] OR "biliary atresia"[tiab] OR "biliary fistula"[tiab] OR "Biliary tract disease*"[tiab] OR "biliary tract cancer*"[tiab] OR "biliary tract neoplasm*"[tiab] OR "bowel obstruction"[tiab] OR Caries[tiab] OR cavities[tiab] OR cavity[tiab] OR "cecal disease*"[tiab] OR "choledochal cyst"[tiab] OR cholelithiasis[tiab] OR "Cholera Infantum"[tiab] OR "cholera morbus"[tiab] OR "chylous ascites"[tiab] OR colitis[tiab] OR "colonic disease*"[tiab] OR "congenital hyperinsulinism"[tiab] OR constipation[tiab] OR "Deglutition Disorder*"[tiab] OR Dental[tiab] OR "diaphragmatic eventration"[tiab] OR diarrhea[tiab] OR diet[tiab] OR dietary[tiab] OR "diverticular disease*"[tiab] OR "duodenal disease*"[tiab] OR "duodenal ulcer*"[tiab] OR "duodenogastric reflux"[tiab] OR dysentery[tiab] OR dysphagia[tiab] OR entercolitis[tiab] OR enteritis[tiab] OR enteropathy[tiab] OR enteropathies[tiab] OR "esophageal atresia"[tiab] OR "esophageal cancer"[tiab] OR "esophageal cyst*"[tiab] OR "esophageal disease*"[tiab] OR "esophageal diverticulosis"[tiab] OR "esophageal fistula*"[tiab] OR "esophageal neoplasm*"[tiab] OR "esophageal perforation"[tiab] OR "esophageal stenosis"[tiab] OR "esophageal varices"[tiab] OR esophagitis[tiab] OR "fatty liver"[tiab] OR feeding[tiab] OR "gallbladder disease*"[tiab] OR "gastric antral vascular ectasia"[tiab] OR "gastric dilatation"[tiab] OR "gastric fistula*"[tiab] OR "gastric outlet obstruction"[tiab] OR "gastric varices"[tiab] OR gastritis[tiab] OR "gastro tube"[tiab] OR "gastro tubes"[tiab] OR gastroenteritis[tiab] OR gastronomy[tiab] OR gastronomies[tiab] OR gastroparesis[tiab] OR gastrostomies[tiab] OR Gastrostomy[tiab] OR gingivitis[tiab] OR glossopalatine[tiab] OR hematemesis[tiab] OR hemoperitoneum[tiab] OR "hepatic infarction"[tiab] OR "hepatic insufficiency"[tiab] OR "hepatic veno-occlusive disease"[tiab] OR hepatitis[tiab] OR "hepatobiliary disease*"[tiab] OR "hepatobiliary disorder*"[tiab] OR "hepatolenticular degeneration"[tiab] OR hepatomegaly [tiab] OR "Hirschsprung disease"[tiab] OR "HIV enteropathy"[tiab] OR "ileal disease*"[tiab] OR incontinence[tiab] OR incontinent[tiab] OR "inflammatory bowel disease*"[tiab] OR IBD[tiab] OR "imperforate anus"[tiab] OR "intestinal atresia"[tiab] OR "intestinal cancer"[tiab] OR "intestinal disease*"[tiab] OR "intestinal failure"[tiab] OR "intestinal fistula*"[tiab] OR "intestinal neoplasm*"[tiab] OR "intestinal obstruction*"[tiab] OR "intestinal perforation"[tiab] OR "intestinal polyposis"[tiab] OR jaw[tiab] OR "jejunal disease*"[tiab] OR kupffer[tiab] OR "lactose intolerance"[tiab] OR "leaky gut"[tiab] OR "levator palati"[tiab] OR "liver abscess*"[tiab] OR "liver cancer*"[tiab] OR "liver cirrhosis"[tiab] OR "liver disease*"[tiab] OR "liver injury"[tiab] OR "liver injuries"[tiab] OR "liver neoplasm*"[tiab] OR malabsorption[tiab] OR malocclusion[tiab] OR malnutrition[tiab] OR mandibular[tiab] OR masticatory[tiab] OR maxillary[tiab] OR "Meckel Diverticulum"[tiab] OR "median arcuate ligament syndrome"[tiab] OR melena[tiab] OR "mesenteric ischemia"[tiab] OR "mesenteric lymphadenitis"[tiab] OR "mesenteric vascular occlusion"[tiab] OR "Migrating Myoelectric complex"[tiab] OR mucositis[tiab] OR "musculus uvulae"[tiab] OR "nutrition issues"[tiab] OR oral[tiab] OR Orodental[tiab] OR palatal[tiab] OR palate[tiab] OR palatoglossus[tiab] OR palatopharyngeus[tiab] OR "pancreas divisum"[tiab] OR "pancreaticobiliary maljunction"[tiab] OR "pancreatic cancer"[tiab] OR "pancreatic cyst"[tiab] OR "pancreatic disease*"[tiab] OR "pancreatic fistula*"[tiab] OR "pancreatic insufficiency"[tiab] OR "pancreatic neoplasm*"[tiab] OR pancreatitis[tiab] OR "peptic ulcer"[tiab] OR "peptic ulcers"[tiab] OR "periodontal bone loss"[tiab] OR "periodontal reabsorption"[tiab:~0] OR "periodontal resorption"[tiab] OR "peritoneal cancer*"[tiab] OR "peritoneal disease*"[tiab] OR "peritoneal fibrosis"[tiab] OR "peritoneal neoplasm*"[tiab] OR "peritoneal panniculitis"[tiab] OR peritonitis[tiab] OR pharyngeal[tiab] OR pharynx[tiab] OR "pneumatosis cystoides intestinalis"[tiab] OR pneumoperitoneum[tiab] OR "postcholecystectomy syndrome"[tiab] OR "postgastrectomy syndrome*"[tiab] OR proctitis[tiab] OR "rectal disease*"[tiab] OR regurgitat*[tiab] OR "rumination syndrome"[tiab] OR salpingopharyngeus[tiab] OR sialorrhea[tiab] OR sialorrhoea[tiab] OR "Sjogren’s Syndrome"[tiab] OR stomachache[tiab] OR "stomach cancer"[tiab] OR "stomach diseases"[tiab] OR "stomach diverticulosis"[tiab] OR "stomach neoplasm*"[tiab] OR "stomach rupture"[tiab] OR "stomach ulcer"[tiab] OR "stomach ulcers"[tiab] OR "stromal tumor*"[tiab] OR "stomach volvulus"[tiab] OR stylopharyngeus[tiab] OR swallow*[tiab] OR teeth[tiab] OR "Temporomandibular Joint disorder"[tiab] OR "tensor palati"[tiab] OR TMJ[tiab] OR toileting[tiab] OR tooth[tiab] OR uvula[tiab] OR "visceral prolapse"[tiab] OR vomit*[tiab] OR "Zellweger syndrome"[tiab] OR "Zollinger-Ellison Syndrome"[tiab] |
| #3 | #1 AND #2 |
| #4 | #3 NOT (Allchild[Filter] NOT Alladult[Filter]) |
| #5 | "Guideline"[pt] OR "practice guideline"[pt] OR "consensus development conference"[pt] OR "consensus development conference, NIH"[pt] OR guideline*[ti] OR standards[ti] OR consensus*[ti] OR recommendat*[ti] OR guideline*[cn] OR standards[cn] OR consensus*[cn] OR recommendat*[cn] OR "practice parameter*"[ti] OR "position statement*"[ti] OR "practice bulletin*"[ti] OR "policy statement*"[ti] OR CPG[ti] OR CPGs[ti] OR "best practice*"[ti] OR (care[ti] AND (path[ti] OR paths[ti] OR pathway[ti] OR pathways[ti] OR map[ti] OR maps[ti] OR plan[ti] OR plans[ti] OR standard[ti])) OR ((critical[ti] OR clinical[ti] OR practice[ti]) AND (path[ti] OR paths[ti] OR pathway[ti] OR pathways[ti] OR protocol*[ti])) OR (algorithm*[ti] AND (pharmacotherap*[ti] OR chemotherap*[ti] OR chemotreatment*[ti] OR therap*[ti] OR treatment*[ti] OR intervention*[ti])) OR (algorithm*[ti] AND (screening[ti] OR examination[ti] OR test[ti] OR tested[ti] OR testing[ti] OR assessment*[ti] OR diagnosis[ti] OR diagnoses[ti] OR diagnosed[ti] OR diagnosing[ti])) OR guideline*[ot] OR standards[ot] OR consensus*[ot] OR recommendat*[ot] OR "practice parameter*"[ot] OR "position statement*"[ot] OR "practice bulletin*"[ot] OR "policy statement*"[ot] OR CPG[ot] OR CPGs[ot] OR "best practice*"[ot] OR (care[ot] AND (path[ot] OR paths[ot] OR pathway[ot] OR pathways[ot] OR map[ot] OR maps[ot] OR plan[ot] OR plans[ot] OR standard[ot])) OR ((critical[ot] OR clinical[ot] OR practice[ot]) AND (path[ot] OR paths[ot] OR pathway[ot] OR pathways[ot] OR protocol*[ot])) OR (algorithm*[ot] AND (pharmacotherap*[ot] OR chemotherap*[ot] OR chemotreatment*[ot] OR therap*[ot] OR treatment*[ot] OR intervention*[ot])) OR (algorithm*[ot] AND (screening[ot] OR examination[ot] OR test[ot] OR tested[ot] OR testing[ot] OR assessment*[ot] OR diagnosis[ot] OR diagnoses[ot] OR diagnosed[ot] OR diagnosing[ot])) OR (("Systematic review"[ti] OR "systematic review"[pt] OR "systematic review"[ot]) AND ("practice guideline*"[tiab] OR "treatment guideline*"[tiab] OR "clinical guideline*"[tiab] OR "guideline recommendation*"[tiab])) |
| #6 | "systematic"[filter] OR "meta-analysis"[pt] OR "meta-analysis as topic"[mh] OR "meta analy*"[tw] OR metanaly*[tw] OR metaanaly*[tw] OR "met analy*"[tw] OR "integrative research"[tiab] OR "integrative review*"[tiab] OR "integrative overview*"[tiab] OR "research integration*"[tiab] OR "research overview*"[tiab] OR "collaborative review*"[tiab] OR "collaborative overview*"[tiab] OR "systematic review"[pt] OR "systematic reviews as topic"[mh] OR "systematic review*"[tiab] OR "technology assessment*"[tiab] OR "technology overview*"[tiab] OR "technology appraisal*"[tiab] OR "Technology Assessment, Biomedical"[mh] OR HTA[tiab] OR HTAs[tiab] OR "comparative efficacy"[tiab] OR "comparative effectiveness"[tiab] OR "outcomes research"[tiab] OR "indirect comparison*"[tiab] OR "Bayesian comparison"[tiab] OR (("indirect treatment"[tiab] OR "mixed-treatment"[tiab]) AND comparison*[tiab]) OR Embase*[tiab] OR Cinahl*[tiab] OR "systematic overview*"[tiab] OR "methodological overview*"[tiab] OR "methodologic overview*"[tiab] OR "methodological review*"[tiab] OR "methodologic review*"[tiab] OR "quantitative review*"[tiab] OR "quantitative overview*"[tiab] OR "quantitative synthes*"[tiab] OR "pooled analy*"[tiab] OR Cochrane[tiab] OR Medline[tiab] OR Pubmed[tiab] OR Medlars[tiab] OR handsearch*[tiab] OR "hand search*"[tiab] OR "meta-regression*"[tiab] OR metaregression*[tiab] OR "data synthes*"[tiab] OR "data extraction"[tiab] OR "data abstraction*"[tiab] OR "mantel haenszel"[tiab] OR peto[tiab] OR "der-simonian"[tiab] OR dersimonian[tiab] OR "fixed effect*"[tiab] OR "multiple treatment comparison"[tiab] OR "mixed treatment meta-analys*"[tiab] OR "umbrella review*"[tiab] OR (("multiple paramet*"[tiab]) AND ("evidence synthesis"[tiab])) OR (("multi-paramet*"[tiab]) AND ("evidence synthesis"[tiab])) OR ((multiparameter*[tiab]) AND ("evidence synthesis"[tiab])) OR "Cochrane Database Syst Rev"[Journal] OR "health technology assessment winchester, england"[Journal] OR "Evid Rep Technol Assess (Full Rep)"[Journal] OR "Evid Rep Technol Assess (Summ)"[Journal] OR "Int J Technol Assess Health Care"[Journal] OR "GMS Health Technol Assess"[Journal] OR "Health Technol Assess (Rockv)"[Journal] OR "Health Technol Assess Rep"[Journal] |
| #7 | (clinical[tiab] AND trial[tiab]) OR "Clinical Trials as Topic"[MeSH] OR "Clinical Trial"[Publication Type] OR random*[tiab] OR "Random Allocation"[MeSH] OR "Therapeutic Use"[Subheading] OR "Multicenter Study"[Publication Type] OR "Multicenter Studies as Topic"[MeSH] OR "controlled trial*"[tiab] OR ((single[tiab] OR doubl*[tiab] OR tripl*[tiab] OR treb*[tiab]) AND (blind*[tiab] OR mask*[tiab])) |
| #8 | "Cohort Studies"[MeSH] OR "Cross Sectional Studies"[MeSH] OR cohort[tiab] OR longitudinal[tiab] OR prospective[tiab] OR retrospective[tiab] OR observational[tiab] OR "cross sectional"[tiab] OR "crosssectional"[tiab] OR "prevalence stud*"[tiab] |
| #9 | #5 OR #6 OR #7 OR #8 |
| #10 | #4 AND #9 |
| #11 | #10 AND (1990/1/1:3000/12/12[pdat]) |
