# Supplementary material for: Gastroenterological disorders and hepatic disease in adults with cerebral palsy: A systematic review
Source: Dev Med Child Neurol. 2025 Oct 30;68(3):313–31. doi: 10.1111/dmcn.70034 (PMC12875176; doi:10.1111/dmcn.70034)
Supplement: Supplementary file 2 — Appendix S2: Eligibility criteria by question. [file DMCN-68-313-s009.docx]

**Appendix S2: Eligibility criteria by question**

| 1. **What is the prevalence and incidence of GI disorders among adults with cerebral palsy and how does it compare to the general adult population, including by decade of life?** | |
| --- | --- |
| **Condition** | Prevalence or incidence of:   - GERD - Constipation - Dysphagia/motor abnormalities with swallowing - Fecal incontinence - Hepatic disease - Dental/oral cavity disorder - Colorectal cancer |
| **Context** | Any country worldwide and any setting (e.g. population-based or hospital-based). |
| **Population** | Adults with CP aged 16 years or older. However, where studies include people aged 16 and 17 years, they must also include adults aged 18 years and older to be included in the review. |
| **Study design** | Cohort studies  Cross-sectional studies  Systematic reviews that directly address the question and were conducted in the last 3 years |

| 1. **Are ambulatory status (i.e. GMFCS I, II III vs IV or V), intellectual disability, subtype of CP, or obesity associated with the incidence or prevalence of the following GI disorders among adults with cerebral palsy?** | |
| --- | --- |
| **Population** | Adults with CP aged 16 years or older. However, where studies include people aged 16 and 17 years, they must also include adults aged 18 years and older to be included in the review. |
| **Exposure** | GMFCS level I-III vs IV/V  Intellectual disability vs no intellectual disability  Subtype of CP (predominantly spastic, dyskinetic, ataxic, including pattern)  BMI (categorised or continuous) |
| **Outcome** | Prevalence or incidence of:   - GERD - Constipation - Dysphagia/motor abnormalities with swallowing - Fecal incontinence - Hepatic disease - Dental/oral cavity disorder - Colorectal cancer |
| **Study design** | Cohort studies  Case-control studies  Cross-sectional studies  Systematic reviews that directly address our question of interest and were conducted in the last 3 years |

| 1. **Are there screening tools that have been used, such as questionnaires or other testing, to assess for the presence, the type and/or the severity of GI disorders in adults with CP, and are these tools valid and feasible in adults with CP?** | |
| --- | --- |
| **Population** | Adults with CP aged 16 years or older. However, where studies include people aged 16 and 17 years, they must also include adults aged 18 years and older to be included in the review. |
| **Instrument** | Patient-reported instruments  Clinician-reported instruments  Laboratory tests  Physical examination |
| **Construct** | - Gastroesophageal reflex disease - Constipation - Dysphagia/motor abnormalities with swallowing - Fecal incontinence - Colorectal cancer |
| **Outcome** | - Validity - Reliability - Responsiveness - Feasibility |
| **Study design** | Any quantitative study design that aims to develop and/or assess the psychometric properties of an instrument  Systematic reviews that directly address our question of interest and were conducted in the last 3 years |

| 1. **Are there interventions identified that can be used for treatment of GI disorders in adults with CP, are they effective in improving symptoms or preventing complications, are the safe, and do they improving HRQOL?** | |
| --- | --- |
| **Population** | Adults with CP aged 16 years or older. However, where studies include people aged 16 and 17 years, they must also include adults aged 18 years and older to be included in the review. |
| **Intervention** | Any intervention that aims to treat the following GI disorders:   - GERD - Constipation - Fecal incontinence - Dysphagia - Hepatic disease - Dental/oral cavity disorder - Colorectal cancer   Interventions may include, but not be limited to, pharmacological interventions, surgical interventions, and therapeutic interventions. |
| **Comparator** | - Usual care - No intervention - A modified version of the intervention - Different intervention - Placebo |
| **Outcome** | - Health-related quality of life (HRQoL) - Symptoms of GERD, constipation, fecal incontinence, dysphagia, hepatic disease, dental/oral cavity disorder - Progression of GERD, constipation, fecal incontinence, dysphagia, hepatic disease, dental/oral cavity disorder - Adverse events |
| **Study design** | RCTs  controlled before- and after-studies  uncontrolled before- and after-studies  interrupted time series  Systematic reviews that directly address our question of interest and were conducted in the last 3 years |

| 1. **Has screening for hepatic diseases or colorectal cancer in adults with CP been demonstrated to allow earlier identification and interventions for either and if present is screening effective in preventing progression, and/or does it lead to improved health-related QOL and do these factors vary by co-morbidity or medication use?** | |
| --- | --- |
| **Population** | Adults with CP aged 16 years or older. However, where studies include people aged 16 and 17 years, they must also include adults aged 18 years and older to be included in the review. |
| **Intervention** | Any screening tool for liver disease or colorectal cancer |
| **Comparator** | - Usual care - No intervention - A modified version of the intervention - Different intervention - Placebo |
| **Outcome** | - Incident hepatic disease - Health-related quality of life (HRQoL) - Progression of liver disease - Adverse events |
| **Study design** | RCTs  Cohort studies  controlled before- and after-studies  uncontrolled before- and after-studies  interrupted time series  Systematic reviews that directly address our question of interest and were conducted in the last 3 years |
