# Supplementary material for: Gastroenterological disorders and hepatic disease in adults with cerebral palsy: A systematic review
Source: Dev Med Child Neurol. 2025 Oct 30;68(3):313–31. doi: 10.1111/dmcn.70034 (PMC12875176; doi:10.1111/dmcn.70034)
Supplement: Supplementary file 4 — Figure S1: PRISMA diagram. [file DMCN-68-313-s007.docx]

Studies from databases/registers **(n = 3783)**

Studies from databases/registers (n = 3733)

Citation searching (n = 50)

**Identification**

Studies included in review **(n = 32)**

Studies excluded **(n = 1630)**

Studies not retrieved **(n = 0)**

Studies assessed for eligibility **(n = 355)**

Studies sought for retrieval **(n = 355)**

Studies screened **(n = 1985)**

Studies excluded **(n = 323)**

Wrong outcome (n = 84)

Wrong study design (n = 129)

Wrong patient population (n = 110)

Duplicates removed **(n = 1798)**

**Screening**

**Included**
