# Supplementary material for: Gastroenterological disorders and hepatic disease in adults with cerebral palsy: A systematic review
Source: Dev Med Child Neurol. 2025 Oct 30;68(3):313–31. doi: 10.1111/dmcn.70034 (PMC12875176; doi:10.1111/dmcn.70034)
Supplement: Supplementary file 5 — Table S1: Description of included studies. [file DMCN-68-313-s005.docx]

**Table S 1 Description of included studies**

| Author (year) | Study design | Country | Years completed | Population (source and eligibility) | Sample size | Age, yr, mean (SD) [min, max] | Female, % | CP subtype, % | GMFCS level,^b^ % | ID, % | Disorder reported |
| --- | --- | --- | --- | --- | --- | --- | --- | --- | --- | --- | --- |
| **Prevalence study** | | | | | | | | | | | |
| Al-Allaq (2014)[^47^](#_ENREF_47) | Cross Sectional | US | 2008-2009 | A random sample of persons with CP who had received care at a specialized dental clinic of a rehabilitation center in California, over a two-year period, were selected from the hospital database. Those included could be living in family home or a group home. Data was categorized by four age groups, 3 of which were adults only, | 323 | 21-35yrs:  26.6 years  36-55 yrs: 44.5 years  ≥ 56 years:  61.3 years | NR | NR | NR | NR | Dental/oral cavity |
| Bell [^27^](#_ENREF_27) (2023) | Cross- sectional | United Kingdom | 2022 | Adults with CP in the UK, aged ≥ 18 years with access to a computer to complete an online survey which was circulated through the University of Nottingham, Scope, the project team’s networks in disability studies, rehabilitation and health sciences, and social media including groups for adults with CP. | 395 | 18-24 yr 7.1%; 35-34 yr 26.1%; 45-54 yr 26.1%;  45-54 yr 18.7%;  55-64 yr 16.2%; ≥65 yr 5.6%;  Prefer not to say 0.3% | Female 74.2%; male 24.8%;  nonbinary 0.8%;  any other gender 0.3% | Spastic 63.8%;  Dyskinetic 6.8%;  Mixed 20.5%;  Not sure/prefer not to say 4.3% | Walk unaided always 23.0%; walk unaided sometimes and sometimes use mobility aid 27.1%; walk aided all the time 7.6%; walk aided sometimes and use a wheelchair at other times 14.2%; use a wheelchair full-time 28.1% | NR | Dysphagia |
| Benigni [^39^](#_ENREF_39) (2011) | Cross-sectional | France | 2004-5 | Practitioners at 15 specialized French institutions for multi-handicapped adults were ask to include CP patients living in these; excluded patients with diagnoses suggesting CP of postnatal origin (traumatic brain injury, near-drowning, motor vehicle accidents, brain tumours or other acquired injuries), patients with significant concomitant diagnoses (vascular cerebral ischemia, neuromuscular diseases, degenerative disorders, autism, psychiatric or behavioural disorders), patients with enteral nutrition and those without a legal guardian’s agreement for blood sampling. | 365 | 35.8 (9.0)  [18-63] | 46% | NR | NR | NR | Constipation  Dental/oral cavity  Dysphagia  GERD |
| Benner [^28^](#_ENREF_28) (2017) | Cross-sectional | Netherlands | NR | Adults previously followed/known to a paediatric rehabilitation centre in the Hague; included those with diagnosis of CP, born between 1965 and 1974, known to the centre and remained in the area. | 49 | 39.8 (3.0)  [35-45] | 45% | Spastic 75% (Unilateral 38%; Bilateral 38%); Non-Spastic 25% | I-III 80%  IV-V 20% | 22%^*^ | Constipation  Fecal incontinence |
| Fortuna [^34^](#_ENREF_34) (2018) | Cross-sectional | US | NR | Adults 18 years and over followed in 35 university-affiliated primary care practices and had ICD 9 codes for cerebral palsy in the electronic medical record. and walking (categorized into Independent, needs assistance or totally dependent) ID categorized as mild-moderate or severe-profound. | 229 | 18-29 yr 24.5%;  30-39 yr 17.9%;  40-49 yr 22.7%;  50-59 yr 17.9%;  ≥60 yr 17.9% | 41% | NR | Walking independently:  18-29 yr 48.2%;  30-39 yr 58.5%;  40-49 yr 62.9%;  50-59 yr 63.4%;  ≥60 yr 40.0% | 54% | Constipation  GERD |
| Henderson [^35^](#_ENREF_35) (2009) | Cross-sectional | US | NR | Identified through state registry of group homes (government or not-for-profit) in two regions of New York state, homes had at least one person with ID aged 40 years or greater Each group home solicited to complete the RHSS on all residents aged ≥40 years (except 4 were less than 40) and walking (categorized into Independent, needs assistance or totally dependent) ID categorized as mild-moderate or severe-profound. | 177 | NR | NR | NR | NR | NR | Dysphagia  GERD |
| Hilberink [^29^](#_ENREF_29)(2007) | Cross-sectional | Netherlands | 1999-2001 | Adults born between 1 Jan 1965 and 31 Dec1974 and known to the rehabilitation centre in the Hague, inclusive of city and surrounding area. | 54 | 30.0 (3.4) | 52% | Spastic paresis 100% (hemiplegia 37%;  diplegia 22%; quadriplegia 41%) | I :28%;  II: 34%;  III: 7%;  IV: 24%;  V: 7% | NR | Dysphagia  Fecal incontinence |
| Jonsson [^30^](#_ENREF_30)(2021) | Cross-sectional | Sweden | 2015-2019 | Adults with CP born in western Sweden 1959-78 and still residing in the area; those who had moved to this population area in Sweden were also invited to participate through patient organizations and habilitation units | 153 | 48.3 (42-55)^a^  [37-58] | 43% | Unilateral spastic 41%;  Bilateral spastic 36%;  Dyskinetic 19%;  Ataxic 4% | I 39%;  II 21%;  III 12%;  IV 17%;  V 11% | 22% | Constipation  Dysphagia |
| Laugharne [^40^](#_ENREF_40)(2024) | Cross-sectional | United Kingdom | 2024 | Survey was sent, by email and/or post, to carers of all adult patients with intellectual disabilities on the caseloads of four intellectual disability services. | 24 | NR | NR | NR | NR | 100% | Constipation |
| Marciniak [^31^](#_ENREF_31)(2015) | Cross-sectional | US | 2009-2011 | Adults 18 and older with diagnosis of CP followed at a rehabilitation hospital approached to participate who were able to provide consent as determined by treating physician | 91 | 36^a^  [18-24] | 49% | NR | I 4%  II 20%  III 13%  IV 42%  V 21% | NR | Constipation  Fecal incontinence |
| Margre  [^24^](#_ENREF_24)(2010) | Cross-sectional | Brazil | NR | Adults 18 and older recruited from three rehabilitation centers and residing in Diamantina, Brazil, with confirmed diagnosis of CP in their medical records. | 22 | 28.7 (10.6)  [18-52] | 36% | Dyskinetic 13.6%;  Spastic quadriplegic 40.9%;  Spastic hemiplegic 22.7%;  Spastic diplegic 22.7% | I:32%  II: 9%  III: 5%  IV: 14%  V: 41% | NR | Dental/oral cavity Dysphagia  GERD |
| Morad [^36^](#_ENREF_36)(2007) | Cross-sectional | Israel | NR | Persons with ID in 60 residential centers at least 40 years old. | 320 | NR | NR | NR | NR | 100% | Constipation |
| Murphy [^46^](#_ENREF_46)(1995) | Cross-sectional | US | NR | Adults with the diagnosis of CP living in the community were recruited through two United Cerebral Palsy Association affiliates of Alameda-Contra Costa counties. | 101 | 42.6  [19-74] | 48% | Spastic 48.5% (hemiparesis 9.9%; quadriparesis 27.7%; diplegia 10.9%);  Dyskinetic (athetosis, chorea or dystonia) 51.5% | NR | NR | Dental/oral cavity |
| Ohwada [^38^](#_ENREF_38)(2006) | Cross-Sectional | Japan | 2001 | Adults with ID residing at public facility in Japan were included. | 90 | Male: 42.1 (10.7)  Female: 43.0 (11.8) | 47% | NR | NR | 100% | GERD  Liver disease |
| Park [^32^](#_ENREF_32)(2018) | Cross-sectional | Korea | 2014 | Adults 19 or older recruited from outpatient clinics or rehab departs or hospitals or national federations for persons with disability, who could complete the questionnaire and CP diagnosis could be confirmed | 154 | 40.2 (9.2) | 40% | Spastic 40.9%; Dyskinetic 20.8%; Ataxic 0.6%; Mixed 30.5%; Do not know/no response 7.1% | I:16.3%  II: 34.8%  III: 7.4%  IV: 34.1%  V: 7.4% | NR | GERD |
| Seo [^22^](#_ENREF_22)(2019) | Cross-sectional | Korea | 2014-2017 | Adults >20 years with dyskinetic CP and cervical dystonia for greater than 1 year who were enrolled in a larger clinical trial evaluating botulinum toxin use for CD in adults with CP were included. | 17 | 47.7 (6.3)  [35--59] | 53% | Dyskinetic 100% | I: 23.5%  II: 41.2%  III: 0.0%  IV: 29.4%  V: 5.9% | NR | Dysphagia |
| Turk [^26^](#_ENREF_26) (1997) | Cross Sectional | US | NR | This was a sample of community dwelling women with CP who were 20 year or older. | 63 | 37.7 (12.7)  [20-74] | 100% | spastic diplegia 29%; dyskinesia/posturing 25%; spastic hemiplegia 18%; other (ataxia, hypotonia, mixed) 16%; spastic quadriplegia 11% | wheelchair user 46% | 34% | Dental/oral cavity  GERD |
| Whitney [^43^](#_ENREF_43) (2019) | Cross-sectional | US | 2016 | Random 20% sample of fee-for service claims from Medicare Part A and B for 18–64 years with ICD 10 codes for CP and 12 months of continuous enrolment in 2016 and “primary race” was White, Black or Hispanic were included. | 16,488 | White  46.3 (11.3);  Black  43.1 (12.3);  Hispanic  37.2 (11.0) | 46% | NR | NR | NR | Liver disease |
| Whitney [^44^](#_ENREF_44) (2019) | Cross-sectional | US | 2016 | Adults 18-64 years and had continuous enrolment in 2016 in the Optum Clinformatics Data Mart Database, a US nationwide deidentified single private payer administrative claims database, with at least one service claim in 2016 were included. | 5,555 | 42.3 (13.9) | 48% | NR | NR | NR | Liver disease |
| Whitney [^41^](#_ENREF_41) (2020) | Cross-sectional | US | 2013 | Adults 18 years or older with CP (identified using ICD-9 code), with complete data for race, without advanced CKD (CKD stage 4 or later), enrolment in a health plan and had 1 or more service utilisation, were identified from the Optum Clinformatics Data Mart Database (OptumInsight Inc), which is a national single private payer administrative claims database containing information from privately insured or Medicare Advantage members. | 8,011 | 49.4 (19.9) | 49.9% | NR | NR | 20.3% | Dysphagia |
| Whitney [^25^](#_ENREF_25)  (2020) | Cross-sectional | US | 2017 | Adults 18 years and older with 12 months of continuous enrolment in 2017 and at least one health care service in the Optum Clinformatics Data Mart Database, a US nationwide deidentified single private payer administrative were enrolled. | 8,077 | CP only: 53.7 (18.0)  CP with NDD: 44.2 (17.3) | 50% | NR | NR | 13% | Constipation  Dysphagia |
| Whitney [^37^](#_ENREF_37) (2021) | Cross-sectional | US | 2014-2016 | Adults who were 18 years or older and with one or more claims identifying CP on two separate days and continuous enrolment in a health plan beginning 2014 in the Optum Clinformatics Data Mart Database, a US nationwide deidentified single private payer administrative claims database were enrolled. | 3,092 | 48.0 (19.2) | 49% | NR | NR | 16% | Dysphagia  Liver disease |
| Whitney[^21^](#_ENREF_21)  (2021) | Cross-sectional | US | 2016 | Adults 18 years or older with CP with more than 1 inpatient claim or more than 2 outpatient claims for CP in 2016, identified from a random 20% sample of fee-for-service Medicare database were identified. Participants had at least 30 days of follow up for mortality and no missing data for demographics | 16,728 | 51.0 (15.3) | 48.2% | NR | NR | 39.3% | Dysphagia  Liver disease |
| Whitney [^23^](#_ENREF_23) (2021) | Cohort | US | 2013-2017 | Adults 18 years and over, without severe CKD or liver disease in 2013 and unbroken enrolment in private health plan; had 1 or more visit to health care provider, and who were then followed 2014 to outcome for mortality, severe CKS, or liver disease, were Identified by ICD 9 or 10 codes | 9,238 | 49.5 (19.8) | 50% | NR | NR | 11% | Liver disease |
| Whitney [^20^](#_ENREF_20) (2021) | Cross-sectional | US | 2016-2018 | Using a 20% random sample of Medicare fee for service claims which included adults at least 18 years and older (65 or older, disabled or on renal dialysis), those with a CP diagnosis were identified by ICD 10 codes for one inpatient or 2 or greater outpatient claims and also had continuous enrolment during 2016 (1 year plus 30 days). | 16,728 | 51.0 (15.3) | 48% | NR | NR | 39% | Dysphagia  Liver disease |
| Whitney[^19^](#_ENREF_19) (2021) | Cross-sectional | US | 2016 | Using a 20% random sample of Medicare fee for service administrative claims database which included adults at least 18 years and older (65 or older, disabled or on renal dialysis), those with a CP diagnosis were identified by ICD 10 codes for one inpatient or 2 or greater outpatient claims. Those included also had continuous enrolment 1 Jan2016-30 Jan2017. | 16,728 | 51.0 (15.3) | 48% | NR | NR | 39% | Dysphagia  Liver disease |
| Whitney [^45^](#_ENREF_45) (2021) | Cross-sectional | US | 2016 | Using 20% random sample of Medicare fee for service administrative claims database which included adults at least 18 years and older (65 or older, disabled or on renal dialysis). CP diagnosis was identified by ICD 10 codes for one inpatient or 2 or greater outpatient claims. All included had continuous enrolment during 2016. | 16,818 | 51.0 (15.3) | 48% | NR | NR | 39% | Liver disease |
| Yi [^33^](#_ENREF_33)  (2019) | Cross-sectional | Korea | 2018 | Investigators interviewed adults on oral diet aged 20 years or older with confirmed CP diagnosis who were recruited from community centers specializing in activities for adults with CP. For inclusion, participants needed to respond to all but 2 items on the Korean Mini mental Status Examination. | 117 | 38.1 (12.4)  [20-79] | 40% | Spastic 42.7%  Dyskinetic 52.1%  Ataxic 2.6%  Mixed 2.6% | I-III:42.7%  IV: 26.5%  V: 30.8% | NR | Dysphagia |
| **Intervention study** | | | | | | | | | | | |
| Bizarra (2020)[^49^](#_ENREF_49) | Randomized Controlled trial | Portugal | NR | Residents of four homes overseen by the Lisbon Cerebral Palsy Association and caregivers working at these homes were invited to participate. Exclusion criteria for residents: Edentulous, unable to cooperate with oral exam and unable to obtain informed consent (person with CP or tutor).  Facilities were randomized to intervention group or no intervention group (2 facilities each group) | 62 | 45 (11.5)  [19-67] | 46.8% | Spastic 80.6%  Tetraplegia 79% | NR | Intervention group: 3.2%  Control group: 32.3% | Dental/oral cavity disorders |
| Cahlin [^48^](#_ENREF_48)  (2019) | Randomized Controlled Trial | Sweden | 2013-2015 | Adults ≥ 18 years, diagnosed with CP and bruxism (diurnal and or nocturnal) and witnessed by relatives and/or caregivers were recruited via hospital dental clinics in the Västra Götaland Region in Sweden. All participants needed to be capable of making decisions and communicating without difficulty, and able to read and understand information. Exclusion criteria were an inability to understand the study and answer questionnaires, sensitivity to botulinum toxin, infections in the injection area, pregnant or breastfeeding, ongoing treatment with botulinum toxin in other body parts, a medication with aminoglycoside antibiotics, spectinomycin, or pharmaceuticals with possible botulinum toxin interaction. | 12 | Intervention (Botulinum toxin A) group 41(13) yrs; control group 44 (17)yrs | 41.7% | NR | NR | 0%** | Dental/oral cavity disorder |
| Davout[^50^](#_ENREF_50) (2016) | Uncontrolled before and after | France | 2005-2013 | Consecutive adult patients with “severe” CP referred to a university hospital for PEG placement under general anesthesia due to swallowing disturbances determined by nasofibroscopy, inadequate oral intake or  recurrent aspiration pneumonia, low weight and including replacement of tube for these reasons. Excluded adults with progressive brain disturbances, general anesthesia ruled out as impossible, and PEG-tube placement refused by guardian. | 19 | 28 [19-48] | 47% | Spastic quadriplegia 94.7%;  Dyskinetic quadriplegia 5.3% | V: 100% | 100% | Dysphagia |
| Pingel[^51^](#_ENREF_51)  (2022) | Uncontrolled before and after | Denmark | NR | NR | 10 | 34.4 (12.3) | 30% | Spastic 100% | I: 20%  II: 10%  III: 30%  V: 40% | NR | Dysphagia |

GERD: Gastroesophageal reflux disease; GMFCS: Gross Motor Function Classification System; ID: Intellectual Disability; NR: Not reported; NDD: neurodevelopmental disabilities; SD: Standard Deviation

^a^Median (interquartile range)

^b^Mobility status as reported if GMFCS level not reported

^*^Assessed by type of education provided. **Assessed by capability of decision making for informed consent and able to read and understand information
