# Supplementary material for: Gastroenterological disorders and hepatic disease in adults with cerebral palsy: A systematic review
Source: Dev Med Child Neurol. 2025 Oct 30;68(3):313–31. doi: 10.1111/dmcn.70034 (PMC12875176; doi:10.1111/dmcn.70034)
Supplement: Supplementary file 6 — Table S2: Summary of findings: prevalence of gastrointestinal disorders and hepatic disease among adults with cerebral palsy. [file DMCN-68-313-s002.docx]

**Table S2 Summary of findings: prevalence of gastrointestinal disorders and hepatic disease among adults with cerebral palsy**

| **Author (year)** | **Assessment of disorders or disease** | **Sample size** | **Context** | **GERD,**  **n (%)** | **Constipation, n (%)** | **Dysphagia,**  **n (%)** | **Fecal incontinence, n (%)** | **Hepatic disease,**  **n (%)** | **Dental/oral cavity disorder,**  **n (%)** |
| --- | --- | --- | --- | --- | --- | --- | --- | --- | --- |
| Al-Allaq (2014)[^47^](#_ENREF_47) | Data extracted from documentation by dental providers (dental residents and attending dentists) which included diagnoses (e.g. caries, periodontitis) and procedures for dental visit. | 323 | Clinic based | NR | NR | NR | NR | NR | Absence of one or more teeth: 21-35 yr 17.6%; 36-55 yr 32.4%; 56+ yrs 59.1%;  All (21-56+ groups): 25%  Excessive Attrition:  21-35 yr 1.6%; 36-55 yr 1.9%; 56+ yrs 4.5%  Periodontal Disease:  21-35 yr 0.5%; 36-55 yr 0.0%; 56+ yrs 0.0%  Anodontia:  21-35 yr 0.5%; 36-55 yr 3.7%; 56+ yrs 0.0%  Tooth eruption Disturbance:  21-35 yr 4.1%; 36-55 yr 4.6%; 56+ yrs 0.0%  Dental Disorder and Disease of Hard Tissues:  21-35 yr 3.1%; 36-55 yr 2.8%; 56+ yrs 4.5%  Partial and complete edentulism:  21-35 yr 1.6%; 36-55 yrs: 13.0%  56+ yrs: 9.1%  Malocclusion and Teeth Crowding:  21-35 yr 1.6%; 36-55 yr 1.9%; 56+ yrs 0.0% |
| Bell[^27^](#_ENREF_27)  (2023) | Adults or proxy (7%) completed online survey with items related to swallowing taken from the pharyngeal symptoms section of the Swallowing Quality of Life Questionnaire (SWAL-QOL). Enteral feeding use was not reported. | 395 | Community online survey | NR | NR | Problems with choking on food - Sometimes, often or almost always: 35%  Problems with coughing out food or liquid- Sometimes, often or almost always: 50.1%.  Problems with coughing out food or liquid by age group-  18–34 yrs: 545 (41.2%)  35–54 yrs: 935 (52.5%)  ≥55 yrs:  51 (59.3%); (*p*<0.05) | NR | NR | NR |
| Benigni[^39^](#_ENREF_39)  (2011) | Charts reviewed using questionnaire developed by practitioner to assess for health conditions using the following definitions. Described malnutrition using Buzby index and assessed other parameters: Gastroesophageal reflux: Treated or suspected; Constipation: untreated or refractory to treatment, Absence of stool lasting over 3 days or hard stools or alternating hard and soft stools; Dysphagia: Coughing during eating or drinking; Poor or severe orodental status: mycoses and mouth dryness, periodontitis, tartar, tooth loss and cavities. Enteral feeding use was a study exclusion. | 365  58% malnutrition | Residential facility | 114 (31.2%) | 60 (16.4%) | 97 (26.5%) | NR | NR | 195 (53.4%) |
| Benner[^28^](#_ENREF_28) (2017) | Constipation: Asked participants if they had regular constipation; Fecal incontinence: derived from participants ratings on the Barthel index, fecal incontinence score, which was dichotomized to yes/no | 31 | Clinic  based | NR | 5 (16.1%) | NR | 2 (6.5%) | NR | NR |
| Fortuna [^34^](#_ENREF_34) (2018) | GERD and Constipation: Medical record review by nurses was used to identify health conditions and function in persons with CP in 35 university-affiliated primary care practices, using the Rochester Health Status Survey. | 229 | Clinic Based | “n” not reported  Total: 42.4%;  18-29 yrs: 41.1%;  30-39 yrs: 34.1%; 40-49 yrs: 41.2%;  50-59 yrs: 39.0%;  >59 yrs: 57.5% | “n” not reported  Total: 4.4%;  18-29 yr 3.6%;  30-39 yr 4.9%;  40-49 yr 2.0%;  50-59 yr 7.3%; >59 yr 5.0% | NR | NR | NR | NR |
| Henderson [^35^](#_ENREF_35) (2009) | Reviewed records of group home (government or private not-for-profit) residents in two regions of New York State. Using the Rochester Health Status survey, nursing staff or service coordinators reviewed medical records and recorded information about diagnoses, health disorders, and skill with ADLS. Enteral feeding use was not reported. | 177 | Residential Facility Based | 21 (12%) | NR | 11 (6%) | NR | NR | NR |
| Hilberink [^29^](#_ENREF_29) (2007) | Physical exam and semi-structured interview performed by physiotherapist. Participants assessed for several health issues and medications reviewed. The use of enteral feedings was not reported. | 54 | Clinic based | NR | NR | 19% | 6% | NR | NR |
| Jonsson [^30^](#_ENREF_30) (2021) | Questionnaires completed by participants who were also interviewed, had physical assessments by multi-professional group experienced with care of those with cerebral palsy. Manual review of medical records was performed. | 153 | Population based | NR | 39  (25.5%) | 45 (29.4%) | NR | NR | NR |
| Laugharne [^40^](#_ENREF_40) (2024) | Questionnaires sent to caregivers of persons with intellectual disabilities with questions designed to detect symptoms of constipation and risk factors, including: bowel movements, use of laxatives and diagnoses including CP. Constipation was defined as ≤ 2 bowels movements per week or regular laxatives ≥ 3 times per week. | 24 | Clinic  based | NR | 16 (66.7%) | NR | NR | NR | NR |
| Marciniak [^31^](#_ENREF_31) (2015) | Research assistant interviewed subjects using validated questionnaires to rate GMFCS level and confirmed with records. Rome III criteria for chronic constipation was used to define if chronic constipation present. International Consultation of Incontinence Questionnaire-Bowel used to rate frequency of bowel control (rating scale: never, rarely sometimes most times or always) and interference of bowel movements with QOL | 91 | Clinic based | NR | 59 (64.8%) | NR | 26 (28.6%) rarely or never had control of formed stool  28 (30.8%) rarely or never control of liquid stool | NR | NR |
| Margre [^24^](#_ENREF_24) (2010) | Participants completed questionnaire and information/diagnoses confirmed with medical record or exam | 22 | Population based | 4 (18.2%) | NR | 9 (40.9%) | NR | NR | 11 (50.0%) |
| Murphy [^46^](#_ENREF_46) (1995) | History and examination performed by primary author to obtain medical diagnoses | 101 | Population based | NR | NR | NR | NR | NR | 27 (26.7%) |
| Ohwada [^38^](#_ENREF_38) (2006) | Retrospective review of health records of persons with ID residing in a residential facility. | 90 | Residential Facility based | 3 (3.3%) | NR | NR | NR | 1 (1.1%) | NR |
| Park [^32^](#_ENREF_32) (2018) | Structured questionnaires about medical and functional status were completed. | 154 | Clinic based | Total:  14 (9.1%);  20-29 yrs:  2 (11.8%);  30-39 yrs:  2 (3.4%);  40-49 yrs:  8 (13.8%);  >50 yrs:  2 (10.0%) | NR | NR | NR | NR | NR |
| Seo [^22^](#_ENREF_22)  (2019) | Subjects were assessed on a clinical dysphagia scale by a physiatrist and history obtained regarding aspiration symptoms from the patient or caregiver. Video fluoroscopic swallow study (VFSS) was performed using a modification of the Logemann protocol. | 17 | NR | NR | NR | CLINICAL ASSESSMENT  -Inadequate chewing:  10 (58.8%)  - Aspiration symptoms during previous week:  3 (17.6%);  - Reflex cough with swallowing 3mL water:  3 (17.6%)  VFSS FINDINGS (SELECTED)  ORAL MOTOR  - Abnormal mastication:  13 (76.5%)  - Premature bolus loss:  10 (58.8%);  PHARYNGEAL PHASE  ABNORMALITIES:  Penetration or aspiration:  10 (58.8%);  - Vallecular residue:  10 (58.8%);  - Laryngeal elevation impaired:  7 (41.2%)    CLINICAL ASSESSMENT  vs VFSS: Subglottic aspiration of thin liquids:  8 (47.1%)  vs clinical symptoms in 4 (23.5%) | NR | NR | NR |
| Turk [^26^](#_ENREF_26) (1997) | Community dwelling women were interviewed and examined. Medical records and information on selected diagnostic testing was reviewed. Secondary conditions were included, based on conditions specific to CP as reported in the Secondary Disability Surveillance System developed by Seekins and colleagues. Dental/oral cavity disorder was indicated by “Poor oral health” | 63 | Community based | 17 (28%) | NR | NR | NR | NR | 43% |
| Whitney [^43^](#_ENREF_43) (2019) | Medicare A and B claims identified by both inpatient and outpatient ICD codes for liver diseases. | 16,488 | Population based | NR | NR | NR | NR | “n” not reported  White 4.7%; Black 3.7%; Hispanic 5.2% | NR |
| Whitney [^44^](#_ENREF_44) (2019) | Medical conditions were identified by searching for specific International Classification of Diseases, 10th Revision, Clinical Modification (ICD-10-CM) codes that are attached to individual claims; a 20% random sample of the Medicare fee-for-service administrative claims data source was evaluated. Liver disease identified by presence of ICD 10 (K 70-77) attached to at least a single claim in the database | 5,555 | Population based | NR | NR | NR | NR | “n” not reported  4.1% | NR |
| Whitney [^41^](#_ENREF_41) (2020) | Medical conditions were identified using 2 or medical claims for conditions coded with ICD 9 codes | 8,011 | Population based | NR | NR | 496 (6.2%) | NR | NR | NR |
| Whitney [^25^](#_ENREF_25) (2020) | Medical conditions were identified by searching for specific International Classification of Diseases, 10th Revision, Clinical Modification (ICD-10-CM) codes that were attached to individual claims; 20% random sample of the Medicare fee-for-service administrative claims data source was used. | 8,077 | Population based | NR | 843 (10.4%) | 835 (10.3%) | NR | NR | NR |
| Whitney [^19^](#_ENREF_19) (2021) | All medical conditions were identified using the International Classification of Diseases, Ninth Revision, Clinical Modification (ICD-9 CM) codes given the time frame of the study and use of ICD code versions in the USA | 3,092 | Population based | NR | NR | 408 (13.2%) | NR | 122 (4.0%) | NR |
| Whitney [^21^](#_ENREF_21)  (2021) | Medical co-morbidities were identified from Medicare administrative claims data used for billing reimbursement utilizing identified by ICD-10-CM attached to the claims. | 16,728 | Population based | NR | NR | 3489  (20.9%) | NR | 799 (4.8%) | NR |
| Whitney [^23^](#_ENREF_23) (2021) | The outcome measures of severe CKD and liver disease from 2014-2017 used both ICD-9 and ICD-10 codes to account for the shift in reporting codes on October 1, 2015. Liver disease defined by at least 1 claim, which included liver necrosis/failure, non-alcoholic chronic liver disease, non-alcoholic fibrosis/cirrhosis, biliary cirrhosis, chronic hepatitis, or “other” or “unspecified” liver disease. | 9,238 | Population based | NR | NR | NR | NR | 586 (6.3%) | NR |
| Whitney [^20^](#_ENREF_20)  (2021) | Medical conditions were identified by searching for specific International Classification of Diseases, 10th Revision, Clinical Modification (ICD-10-CM) codes that are attached to individual claims; 20% random sample of the Medicare fee-for-service administrative claims data source. | 16,728 | Population based | NR | NR | “n” not reported  20.9 % | NR | “n” not reported  4.8% | NR |
| Whitney [^19^](#_ENREF_19) (2021) | Medical conditions were identified by searching for specific ICD-10-CM codes attached to individual patient claims in a 20% random sample of a Medicare fee for service database. ICD-10-CM codes were used to identify each medical condition. | 16,728 | Population based | NR | NR | “n” not reported  20.9 % | NR | “n” not reported  4.8% | NR |
| Whitney [^45^](#_ENREF_45)  (2021) | Medical conditions were identified by searching for specific International Classification of Diseases, 10th Revision, Clinical Modification (ICD-10-CM) codes that are attached to individual claims; 20% random sample of the Medicare fee-for-service administrative claims data source. | 16,818 | Population based | NR | NR | NR | NR | “n” not reported  18-30 yrs: 3.4%;  31-40 yrs: 4.5%;  41-50 yrs: 5.3%;  51-60 yrs: 6.3%;  61-70 yrs: 6.1%;  71-80 yrs: 6.4%;  >80 yrs:  6.0% | NR |
| Yi [^33^](#_ENREF_33)  (2019) | Adults with CP were interviewed and examined. Physician evaluated functional status and classification of those with CP.  The Swallowing Quality of Life, a prevalence subscale for which the participant rates the component on the oral dysphagia subscale section that indicates frequency of drooling was used for drooling prevalence. The participant are asked to quantifying prevalence of drooling and it was considered present if a rating was chosen of either “sometimes, often or almost always”.  The Functional Oral Intake scale (FOIS) was used for pharyngeal dysphagia ratings, performed by an occupational therapist with swallowing therapy experience who interviewed participant or caregiver. No subjects had enteral tubes. | 117 | Clinic based | NR | NR | Choking on food (sometimes or more) 90 (76.9%)  Chewing problems (sometimes or more) 70 (59.8%)  Oral/pharyngeal dysphagia: Drooling (Sometimes or more) 53 (45.3%) | NR | NR | NR |

CP: Cerebral palsy; CKD: Chronic kidney disease; GMFCS: Gross Motor Function Classification System;

GERD: Gastroesophageal reflux disease; ICD: International Classification of Diseases; ID: Intellectual disability; n: number; NR: Not Reported;

PARD: Speech language hearing dysphagia risk assessment protocol (PARD is the abbreviation for the title in Portuguese)

VFSS: Video Fluoroscopic Swallow Study; Yrs: Years
