# Supplementary material for: Gastroenterological disorders and hepatic disease in adults with cerebral palsy: A systematic review
Source: Dev Med Child Neurol. 2025 Oct 30;68(3):313–31. doi: 10.1111/dmcn.70034 (PMC12875176; doi:10.1111/dmcn.70034)
Supplement: Supplementary file 7 — Table S3: Quality appraisal of prevalence studies. [file DMCN-68-313-s019.docx]

**Table S3: Quality appraisal of prevalence studies**

| Study | Was the sample frame appropriate to address the target population? | Were study participants sampled in an appropriate way? | Was the sample size adequate? | Were the study subjects and the setting described in detail? | Was the data analysis conducted with sufficient coverage of the identified sample? | Were valid methods used for the identification of the condition (GI outcome)? | Was the condition (GI outcome) measured in a standard, reliable way for all participants? | Was there appropriate statistical analysis? | Was the response rate adequate, and if not, was the low response rate managed appropriately? |
| --- | --- | --- | --- | --- | --- | --- | --- | --- | --- |
| Al-Allaq^47^ | yes | yes | no | no | yes | yes | yes | no | not applicable |
| Bell^27^ | no | yes | yes | no | yes | no | yes | yes | unclear |
| Benigni ^39^ | no | unclear | no | no | yes | yes | yes | no | unclear |
| Benner ^28^ | yes | yes | no | yes | yes | unclear | yes | no | yes |
| Fortuna ^34^ | yes | yes | no | no | yes | yes | yes | no | yes |
| Henderson ^35^ | no | no | no | no | yes | no | yes | no | yes |
| Hilberink ^29^ | no | yes | no | yes | yes | unclear | yes | no | yes |
| Jonsson ^30^ | yes | unclear | no | yes | unclear | unclear | yes | yes | unclear |
| Laugharne ^40^ | no | no | no | no | yes | yes | yes | yes | no |
| Marciniak ^31^ | no | yes | no | no | yes | yes | yes | yes | unclear |
| Margre ^24^ | no | unclear | no | yes | yes | no | yes | yes | unclear |
| Murphy ^46^ | yes | no | no | no | yes | unclear | yes | yes | unclear |
| Ohwada ^38^ | no | unclear | no | no | yes | unclear | unclear | yes | unclear |
| Park ^32^ | yes | unclear | no | yes | no | yes | yes | no | yes |
| Seo ^22^ | no | no | no | no | yes | yes | yes | yes | yes |
| Turk ^26^ | no | yes | no | yes | yes | unclear | yes | yes | unclear |
| Whitney ^43^ | yes | yes | yes | no | yes | yes | yes | no | yes |
| Whitney Kamdar Ng ^44^ | yes | yes | yes | no | yes | yes | yes | no | yes |
| Whitney Oliverio Kamdar ^41^ | yes | yes | yes | no | yes | yes | yes | yes | yes |
| Whitney Schmidt Peterson Haapala ^23^ | yes | yes | yes | no | yes | yes | yes | yes | yes |
| Whitney Kamdar ^37^ | yes | yes | yes | no | yes | yes | yes | yes | yes |
| Whitney Oliverio ^21^ | yes | yes | yes | no | yes | yes | yes | yes | yes |
| Whitney Schmidt Haapala ^23^ | yes | yes | yes | no | yes | yes | yes | yes | yes |
| Whitney Schmidt Hurvitz ^20^ | yes | yes | yes | no | yes | yes | yes | no | yes |
| Whitney Basu ^19^ | yes | yes | yes | no | yes | yes | yes | no | yes |
| Whitney Schmidt Haapala Ryan Hurvitz ^45^ | yes | yes | yes | no | yes | yes | yes | yes | yes |
| Yi ^33^ | no | yes | no | yes | yes | yes | yes | yes | yes |
