# Supplementary material for: Gastroenterological disorders and hepatic disease in adults with cerebral palsy: A systematic review
Source: Dev Med Child Neurol. 2025 Oct 30;68(3):313–31. doi: 10.1111/dmcn.70034 (PMC12875176; doi:10.1111/dmcn.70034)
Supplement: Supplementary file 8 — Table S4: Quality appraisal of studies comparing prevalence between adults with and without CP. [file DMCN-68-313-s017.docx]

**Table S4:  Quality appraisal of studies comparing prevalence between adults with and without CP**

| Study | Were the criteria for inclusion in the sample clearly defined? | Were the study subjects and the setting described in detail? | Were objective, standard criteria used for measurement of the condition? | Were confounding factors identified? | Were strategies to deal with confounding factors stated? | Were the outcomes measured in a valid and reliable way? | Was appropriate statistical analysis used? |
| --- | --- | --- | --- | --- | --- | --- | --- |
| Fortuna ^34^ | yes | no | yes | yes | yes | yes | yes |
| Henderson ^35^ | no | no | no | no | no | yes | yes |
| Morad ^36^ | yes | no | no | no | no | yes | no |
| Whitney Kamdar ^37^ | yes | no | yes | yes | yes | yes | yes |
