# Supplementary material for: Gastroenterological disorders and hepatic disease in adults with cerebral palsy: A systematic review
Source: Dev Med Child Neurol. 2025 Oct 30;68(3):313–31. doi: 10.1111/dmcn.70034 (PMC12875176; doi:10.1111/dmcn.70034)
Supplement: Supplementary file 9 — Table S5: Quality appraisal of cross‐sectional studies examining prognostic factors for GI and hepatic disorders. [file DMCN-68-313-s018.docx]

**Table S5: Quality appraisal of cross-sectional studies examining prognostic factors for GI and hepatic disorders**

| Study | Were the criteria for inclusion in the sample clearly defined? | Were the study subjects and the setting described in detail? | Was the exposure measured in a valid and reliable way? | Were confounding factors identified? | Were strategies to deal with confounding factors stated? | Were the outcomes measured in a valid and reliable way? | Was appropriate statistical analysis used? |
| --- | --- | --- | --- | --- | --- | --- | --- |
| Fortuna ^34^ | yes | no | yes | yes | yes | yes | yes |
| Jonsson ^30^ | yes | yes | yes | no | no | yes | no |
| Marciniak ^31^ | yes | no | yes | no | no | yes | no |
| Turk ^26^ | yes | yes | yes | no | no | yes | no |
| Whitney Schmidt Hurvitz ^20^ | yes | yes | yes | yes | yes | yes | no |
| Whitney Basu ^19^ | yes | yes | yes | yes | yes | yes | no |
| Whitney Schmidt Haapala ^23^ | yes | no | yes | yes | yes | yes | yes |
| Seo ^22^ | yes | yes | yes | yes | yes | yes | yes |
