# Supplementary material for: Gastroenterological disorders and hepatic disease in adults with cerebral palsy: A systematic review
Source: Dev Med Child Neurol. 2025 Oct 30;68(3):313–31. doi: 10.1111/dmcn.70034 (PMC12875176; doi:10.1111/dmcn.70034)
Supplement: Supplementary file 12 — Table S8: Summary of clinical evidence profile for prevalence studies. [file DMCN-68-313-s003.docx]

**Table S8 Summary of clinical evidence profile for prevalence studies**

| Outcome | Prevalence findings | Number of participants (studies) | Certainty in the evidence (GRADE) |
| --- | --- | --- | --- |
| Gastrointestinal Reflux Disease prevalence was assessed by a history or medical record review | Prevalence varied by methodology and population studied.  Prevalence rates ranged from 3-42%. | 1,100 adults with CP,  7 observational trials | Low  (due to methodological limitations, imprecision and inconsistency) |
| Constipation prevalence was assessed by history/interview, medical record review, symptoms reported through standardized questionnaire or ICD 10 codes in medical claims database. | Prevalence varied by methodology and population studied. Prevalence rates ranged from 4-67%. | 8,970 adults with CP,  7 observational studies | Low  (due to methodological limitations, imprecision and inconsistency) |
| Dysphagia prevalence was assessed by history, standardized clinical swallow evaluation, medical record review, and/or videoflouroscopic swallow study. | Prevalence varied by methodology and population studied. Prevalence rates ranged from 6-77%. | 37,208 adults with CP, 12 observational studies | Low  (due to methodological limitations, imprecision, and inconsistency) |
| Fecal Incontinence prevalence was  assessed by interview/history using semi-structured interviews or standardized questionnaires, | Prevalence varied by population studied and methodology. Prevalence rates ranged from 6-29%. | 176 adults with CP, 3 observational studies | Low  (due to methodological limitations, imprecision, and inconsistency) |
| Hepatic Disease prevalence was assessed by medical chart review or using ICD 9/10 codes documented in medical claims databases. | Prevalence varied by methodology, population studied, and comorbidities. Prevalence rates ranged from 1-6%. | 68,009 adults with CP, 7 observational studies | Low  (due to methodological limitations and inconsistency) |
| Dental/Oral cavity disorders prevalence was assessed by history, medical record review, and physical examination | Prevalence varied by methodology and population. Prevalence rates ranged from 25-53.4%. | 874 adults with CP, 5 observational studies | Low  (due to methodological limitations, imprecision and inconsistency) |
| Colorectal Cancer | No studies identified for adults with CP that assessed colorectal cancer rate prevalence. | 0 | Absent - prevalence in adults only was not found |

Note: Information by study is presented in Main Study Table 2
