# Supplementary material for: Gastroenterological disorders and hepatic disease in adults with cerebral palsy: A systematic review
Source: Dev Med Child Neurol. 2025 Oct 30;68(3):313–31. doi: 10.1111/dmcn.70034 (PMC12875176; doi:10.1111/dmcn.70034)
Supplement: Supplementary file 13 — Table S9: Summary of clinical evidence profile for studies comparing those with CP versus those without CP. [file DMCN-68-313-s016.docx]

**Table S9 Summary of clinical evidence profile for studies comparing those with CP versus those without CP**

| **Outcome** | **Comparative risk** | **Number of participants (studies)** | **Certainty in the evidence (GRADE)** |
| --- | --- | --- | --- |
| Gastrointestinal Reflux disease was assessed by medical record review. | One study reported that the prevalence in adults with CP was higher than in those without CP, but at living in the same residential facilities. | 77 adults with CP (one observational study) were compared with 1196 individuals without CP | Very low (due to methodological limitations, imprecision and inconsistency) |
| Constipation was assessed by medical record review for adults with CP. | One study reported the prevalence of constipation was lower in adults with CP followed by university-affiliated group practices in a region of New York state, with constipation prevalence lower when compared to the general population across all age groups using population data from the National Health and Nutrition Examination Survey (NHANES) 2009-2010.  The other study reported prevalence in adults with CP living in residential facilities and constipation prevalence was significantly higher in those with CP compared to adults without CP living in these same facilities. | 549 adults with CP (2 observational studies)^a^ | Low (due to methodological limitations and inconsistency) |
| Dysphagia was assessed by medical record review. | One study reported the prevalence of dysphagia was higher in adults with CP compared to those without CP living in the same facilities. | 177 adults with CP across 1 observational study were compared to 1196 individuals without CP. | Very low (due to methodological limitations, imprecision and inconsistency) |
| Hepatic Disease was identified using ICD 9/10 codes for liver disease in a medical claims database. | One study found that the prevalence of hepatic disease was higher in adults with CP compared to those without CP. | 5,555 adults with CP in a de-identified medical claims data base were compare to  5,531,366 without CP in the same database. | Low (due to methodological limitations of the study and inconsistency) |

^a^Number of adults without CP unknown

Note: Information by study is presented in Main Study Table
