# Supplementary material for: Gastroenterological disorders and hepatic disease in adults with cerebral palsy: A systematic review
Source: Dev Med Child Neurol. 2025 Oct 30;68(3):313–31. doi: 10.1111/dmcn.70034 (PMC12875176; doi:10.1111/dmcn.70034)
Supplement: Supplementary file 14 — Table S10: Summary of clinical evidence profile for comparison: GMFCS levels I, II, III, IV, and V or ambulatory vs not ambulatory. [file DMCN-68-313-s015.docx]

**Table S10: Summary of clinical evidence profile for comparison: GMFCS levels I, II, III, IV and V or ambulatory vs Not ambulatory**

| Outcome | Illustrative comparative risk | Number of participants (studies) | Certainty in the evidence (GRADE) |
| --- | --- | --- | --- |
| Gastroesophageal reflux disease as assessed through medical record review | Prevalence in adults who walked independently or mostly independently was lower for GERD prevalence compared to those that were not ambulatory. | 229 adults (1 observational study) | Very low  (due to methodological limitations, imprecision and inconsistency) |
| Constipation prevalence as assessed through medical record or interview using standardized questionnaire. | Prevalence of constipation by interview was higher in one study with increasing GMFCS levels. One other study found constipation prevalence was not significantly different for those levels I-III and IV-V when using a standardized questionnaire. | 224 adults with CP (1 observational study) | Very low  (due to methodological limitations, imprecision and inconsistency) |
| Dysphagia prevalence assess through interview and exam, and videofloroscopic swallow study | Prevalence for dysphagia increased with increasing GMFCS levels on one study. The other study only included those with dyskinetic CP and using videoflouroscopic studies, did not find an association with GMFCS levels. | 170 adults with CP (2 observational studies) | Very low  (due to methodological limitations, imprecision and inconsistency) |
| Fecal incontinence assessed through medical record review, history and interview using standardized questionnaire | Prevalence for fecal incontinence was greater for those less ambulatory (GMFCS IV-V) compared to those levels I-III. | 91 adults (1 observational study) | Very low  (due to methodological limitations, imprecision and inconsistency) |

Note: Information by study is presented in Main Study Table 4
