# Supplementary material for: Gastroenterological disorders and hepatic disease in adults with cerebral palsy: A systematic review
Source: Dev Med Child Neurol. 2025 Oct 30;68(3):313–31. doi: 10.1111/dmcn.70034 (PMC12875176; doi:10.1111/dmcn.70034)
Supplement: Supplementary file 15 — Table S11: Summary of clinical evidence profile for comparison: CP subtype. [file DMCN-68-313-s004.docx]

**Table S11 Summary of clinical evidence profile for comparison: CP subtype**

| Outcome | Illustrative comparative risk | Number of participants (studies) | Certainty in the evidence (GRADE) |
| --- | --- | --- | --- |
| Constipation prevalence was assessed through medical record review, assessments and interviews. | Prevalence of constipation differed by CP subtype, with greater apparent prevalence in those of dyskinetic and ataxic CP subtypes | 153 adults with CP (1 observational study) | Very low  (due to methodological limitations, imprecision and inconsistency) |
| Dysphagia prevalence was assessed through interviews, assessments, and medical record review, | Prevalence for dysphagia differed by CP subtype. Dysphagia prevalence was highest in those with dyskinetic CP. | 153 adults with CP (1 observational study) | Very low  (due to methodological limitations, imprecision and inconsistency) |

Note: Information by study is presented in Main Study Table 4
