# Supplementary material for: Gastroenterological disorders and hepatic disease in adults with cerebral palsy: A systematic review
Source: Dev Med Child Neurol. 2025 Oct 30;68(3):313–31. doi: 10.1111/dmcn.70034 (PMC12875176; doi:10.1111/dmcn.70034)
Supplement: Supplementary file 16 — Table S12: Summary of clinical evidence profile for comparison: Intellectual Disability. [file DMCN-68-313-s013.docx]

**Table S12: Summary of clinical evidence profile for comparison: Intellectual Disability**

| Outcome | Illustrative comparative risk | Number of participants (studies) | Certainty in the evidence (GRADE) |
| --- | --- | --- | --- |
| Gastroesophageal reflux disease as assessed through interview | Prevalence for GERD was significantly associated with ID, however no effect estimate reported | 58 adults with CP (1 observational study) | Very low  (due to methodological limitations, imprecision and inconsistency) |
| Constipation prevalence as assessed interview, exam, and medical record review | Prevalence for constipation was higher for those with ID (IQ <70) vs those without ID. | 153 adults with CP (1 observational study) | Very low  (due to methodological limitations, imprecision and inconsistency) |
| Dysphagia prevalence assessed through interview and exam, and medical record review, or ICD 10 codes in medical claims database. | Two studies reported prevalence for dysphagia was higher for those with ID vs those without ID. | 16,881 adults with CP (2 observational studies) | Moderate (due to methodological limitations) |
| Hepatic Disease was assessed through ICD-10 codes using medical claims databases. | Prevalence of hepatic disease not different between adults with and without ID | 33,546 adults with CP (2 observational studies) | Moderate (due to methodological limitations) |

Note: Information by study is presented in Main Study Table 4
