# Supplementary material for: Gastroenterological disorders and hepatic disease in adults with cerebral palsy: A systematic review
Source: Dev Med Child Neurol. 2025 Oct 30;68(3):313–31. doi: 10.1111/dmcn.70034 (PMC12875176; doi:10.1111/dmcn.70034)
Supplement: Supplementary file 17 — Table S13: Summary of clinical evidence profile for comparison: Obesity. [file DMCN-68-313-s006.docx]

**Table S13: Summary of clinical evidence profile for comparison: Obesity**

| Outcome | Illustrative comparative risk | Number of participants (studies) | Certainty in the evidence (GRADE) |
| --- | --- | --- | --- |
| Constipation prevalence as assessed using a standardized questionnaire. | Prevalence of constipation was not associated with BMI categories of normal/underweight; over -weight and obese | 91 adults with CP (1 observational study) | Very low  (due to methodological limitations, imprecision and inconsistency) |
| Fecal Incontinence | Prevalence for those reporting incontinence for either solid or liquid stool was not associated with BMI categories of normal/underweight overweight or obese, | 91 adults with CP (1 observational study) | Very low  (due to methodological limitations, imprecision and inconsistency) |

Note: Information by study is presented in Main Study Table 4
