# Supplementary material for: Gastroenterological disorders and hepatic disease in adults with cerebral palsy: A systematic review
Source: Dev Med Child Neurol. 2025 Oct 30;68(3):313–31. doi: 10.1111/dmcn.70034 (PMC12875176; doi:10.1111/dmcn.70034)
Supplement: Supplementary file 18 — Table S14: Summary of clinical evidence profile comparison: non‐pharmacological intervention compared to no intervention or usual care. [file DMCN-68-313-s011.docx]

Table S14. Summary of clinical evidence profile comparison: non-pharmacological intervention compared to no intervention or usual care

| Outcome | Effect | Number of participants (studies) | Certainty in the evidence (GRADE) |
| --- | --- | --- | --- |
| Severity of gum inflammation or gingivitis severity assessed using Gingival Index | Improved at 2- and 6-months post-intervention compared to usual care (effect estimate not reported) | 62 (1 RCT) | Very low (due to methodological limitations, imprecision and inconsistency) |
| Oral hygiene status assessed using simplified Oral Hygiene Index (OHI-S) | Improved at 2- and 6-months post-intervention compared to usual care (effect estimate not reported) | 62 (1 RCT) | Very low (due to methodological limitations, imprecision and inconsistency) |
| Weight gain: baseline to long-term follow up | Median change: 4.2 kg (IQR 1.0 kg to 8.8 kg; p<0.01)  15/17 (88.2%) participants gained at least 15% body weight | 17 (1 uncontrolled before and after trial) | Very low (due to methodological limitations, imprecision and inconsistency)) |
| Swallow function assessed using self-reported difficulty with supraglottic swallow exercise, self-reported difficulty with normal swallowing exercise, self-reported difficulty with speaking | Improved at 6 months post-intervention (effect estimate not reported) | 10 (1 uncontrolled before and after trial) | Very low (due to methodological limitations, imprecision and inconsistency) |
| Mealtime Eating and Drinking Abilities assessed by the amount of time that the participants needed to complete the self-chosen  meal | No effect | 10 (1 uncontrolled before and after trial) | Very low (due to methodological limitations, imprecision and inconsistency) |
