# Supplementary material for: Gastroenterological disorders and hepatic disease in adults with cerebral palsy: A systematic review
Source: Dev Med Child Neurol. 2025 Oct 30;68(3):313–31. doi: 10.1111/dmcn.70034 (PMC12875176; doi:10.1111/dmcn.70034)
Supplement: Supplementary file 19 — Table S15: Summary of clinical evidence profile comparison: pharmacological intervention compared to no intervention or usual care. [file DMCN-68-313-s010.docx]

**Table S15 Summary of clinical evidence profile comparison: pharmacological intervention compared to no intervention or usual care**

| Outcome | Effect | Number of participants (studies) | Certainty in the evidence (GRADE) |
| --- | --- | --- | --- |
| Pain in jaw, | No effect at 2 weeks and 16 weeks | 12 participants (1 RCT) | Very low (due to methodological limitations, imprecision and inconsistency) |
| Chewing Efficiency | No effect at 2 weeks and 16 weeks | 12 participants (1 RCT) | Very low (due to methodological limitations, imprecision and inconsistency) |
| Oral health assessed using General Oral Health Assessment Index (Swedish Version) | No effect at 2 weeks and 16 weeks | 12 participants (1 RCT) | Very low (due to methodological limitations, imprecision and inconsistency) |
| Bruxism | No effect at 2 weeks and 16 weeks | 12 participants (1 RCT) | Very low (due to methodological limitations, imprecision and inconsistency) |

RCT = Randomized controlled trial

Note: Information by study is presented in Main Study Table 5
